# Chromosomal position shift of a regulatory gene alters the bacterial phenotype

**DOI:** 10.1093/nar/gkv709

**Published:** 2015-07-13

**Authors:** Veneta Gerganova, Michael Berger, Elisabed Zaldastanishvili, Patrick Sobetzko, Corinne Lafon, Michael Mourez, Andrew Travers, Georgi Muskhelishvili

**Affiliations:** 1 Jacobs University Bremen, School of Engineering and Science, Bremen, 28758, Germany; 2 Institut für Hygiene, Universitätsklinikum Münster, Münster, 48149, Germany; 3 Philipps-Universität Marburg, LOEWE-Zentrum für Synthetische Mikrobiologie, Department of Chromosome Biology, Marburg, 35032, Germany; 4 SANOFI/ TSU Infectious Diseases, Toulouse, 31036, France; 5 MRC Laboratory of Molecular Biology, Francis Crick Avenue, Cambridge CB2 0QH, UK

## Abstract

Recent studies strongly suggest that in bacterial cells the order of genes along the chromosomal origin-to-terminus axis is determinative for regulation of the growth phase-dependent gene expression. The prediction from this observation is that positional displacement of pleiotropic genes will affect the genetic regulation and hence, the cellular phenotype. To test this prediction we inserted the origin-proximal *dusB-fis* operon encoding the global regulator FIS in the vicinity of replication terminus on both arms of the *Escherichia coli* chromosome. We found that the lower *fis* gene dosage in the strains with terminus-proximal *dusB-fis* operons was compensated by increased *fis* expression such that the intracellular concentration of FIS was homeostatically adjusted. Nevertheless, despite unchanged FIS levels the positional displacement of *dusB-fis* impaired the competitive growth fitness of cells and altered the state of the overarching network regulating DNA topology, as well as the cellular response to environmental stress, hazardous substances and antibiotics. Our finding that the chromosomal repositioning of a regulatory gene can determine the cellular phenotype unveils an important yet unexplored facet of the genetic control mechanisms and paves the way for novel approaches to manipulate bacterial physiology.

## INTRODUCTION

Transcriptional regulation of the bacterial growth is associated with dynamic structural changes of the unique bacterial chromosome—the nucleoid. The nucleoid structure has to reconcile the requirement of a roughly 1000-fold compaction of the DNA with unimpeded accessibility of the chromosomal loci to the transcription, replication and repair machineries. This implies a high degree of structural organization. Indeed, recent biochemical studies of the nucleoids isolated from classical bacterial model organism *Escherichia coli* (*E. coli*) proposed an intricate granular structure consistent with a branched DNA supercoil (1), whereas genetic studies identified both small topologically isolated domains (2) and extended macrodomains (3). Recent imaging of the growth phase-dependent variations of the folding of the bacterial chromosome revealed multiple small domains and also a global bent filament shape consistent with coiled organization (4). Furthermore, two independent studies, one using the chromosome conformation capture and the other, the spatiotemporal mapping of functional communications between the genomic loci, proposed that the nucleoid is folded as a plectonemic coil with longitudinally interwound chromosomal arms (5,6).

The organization of the nucleoid structure was shown to depend on the binding effects of the small class of highly abundant nucleoid-associated proteins (NAPs), which not only regulate the chromosomal dynamics but also act as global regulators of gene expression (7–10). Changing growth conditions substantially affect the composition of the NAPs (11), which account for the bulk of protein released from isolated nucleoids (12,13). Accordingly, the genomic spatial transcript profiles (14), the boundaries of the chromosomal topological domains (15), as well as the structure of the nucleoid *in vivo* (16) and *in vitro* (17) are affected by mutations of the NAP genes. Furthermore, the NAPs, together with DNA topoisomerases and components of transcription machinery, form an overarching network of interdependent pleiotropic genes coordinating the growth phase-dependent gene expression and DNA topology in *E. coli* (7).

Importantly, it was observed that distinct genetic loci occupy specific positions in the cell according to their physical arrangement in the chromosome (18–21). Moreover, it was found that in *E. coli* in particular, and γ-Proteobacteria in general, the order of genes is highly conserved along the chromosomal replication origin-to-terminus (OriC-Ter) axis (6). Several lines of evidence suggest that chromosomal position is pertinent to genetic expression. First, it was observed that the *E. coli* chromosome demonstrates distinct transcriptionally active and silenced regions, whereby the latter are proposed to serve as organising centres isolating chromosomal domains (22). Secondly, limited diffusion of the messenger RNAs (mRNAs) from their chromosomal transcription sites was observed, suggesting a physical proximity of the site of translation to the cognate gene locus (23). Thirdly, the diffusion of a DNA binding regulatory protein was found to depend on its genomic production site (24). In addition, the gene order along the chromosomal OriC-Ter axis was found to correlate with temporal gene expression during the bacterial growth cycle, such that the genes involved at the early and late stages of the growth are located respectively closer to the chromosomal origin and terminus of replication (6). Finally, a recent study directly showed that the chromosomal position affects the reporter gene expression (25). The prediction from all these observations is that the effects of global regulatory genes, such as, e.g. those of the NAPs, will also depend on their position along the OriC-Ter axis of the plectonemically interwound bacterial chromosome.

In this study, we tested this prediction using the *dusB-fis* operon encoding the global regulator FIS—an abundant NAP transiently expressed during the early exponential phase (26,27). We moved the *dusB-fis* operon from its original location in the vicinity of the OriC, into close proximity to the replication terminus on both arms of the circular *E. coli* chromosome. We demonstrate that as predicted, the chromosomal repositioning of the *fis* gene affects the cellular phenotype.

## MATERIALS AND METHODS

### Generation of strains with dislocated *dusB-fis* operons

The *E. coli* K12 strain CSH50 (*ara D(lac pro) thi rpsL*) was used throughout these experiments. For generation of WTeng, a chloramphenicol cassette was amplified with primers 1 and 2 (Supplementary Table S2) and inserted between the *fis* and *yhdJ* ORFs via the RedE/T homologous recombination system. The entire *fisP-dusB-fis-cat* construct was amplified with primers carrying overhangs for respectively Terminus Left (TL) or Terminus Right (TR) positions (Supplementary Table S2) and was transformed into CSH50 Δ*fis::kan* RedE/T cells. The amplified *fisP-dusB-fis-cat* construct contains both terminators that naturally flank the *fis* operon region. The control strains with dislocated *yfp* reporter gene were generated similarly. The WTeng-yfp strain (kindly provided by Alissa Respet) contains the *fisP-dusB-yfp-cm* construct in the native *fis* locus, yielding a Δ*fis* background. The *fisP-dusB-yfp-cat* construct was amplified with primers carrying overhangs for Terminus Left (TLyfp), Terminus Right (TRyfp) or Middle Left (the primers are listed in Supplementary Table S2) and transformed into CSH50 RedE/T cells. These insertions were made in both *fis^+^* and *fis^−^* backgrounds, the latter generated via classical P1 transduction. The integrity of all inserts with corresponding flanking regions was monitored by sequencing (Eurofins MWG Operon). All the used strains are described in Supplementary Table S3.

### Growth conditions

The movants and the control strains were grown in rich dYT medium either in microtiter plates at 37°C with shaking (900 rpm) or in flasks at 37°C, 150 rpm, unless otherwise stated. These conditions were used for sample collection of protein, mRNA and chromosomal DNA from all the strains throughout the growth cycle at OD_580_ of 0.3, 0.5, transition, early and late stationary phase (24 h after the start of the growth).

### Competitive growth fitness measurements

Overnight (O/N) cultures of all experimental strains were diluted 1:10 000 in fresh medium and grown for 12 h. Subsequently, the TR and TL strain cultures were mixed with either WTeng or Δ*fis* in a 1:1 ratio. The initial mixtures were diluted 1:10 000 in fresh medium and grown for 12 h. After every 12 h the cultures were sampled for plating on selective plates with kanamycin and non-selective solid medium. The mixtures were again diluted 1:10 000 in fresh medium and grown for another 12 h. This dilution/plating cycle was repeated for at least seven times. The percentage of surviving cells was determined from the ratios of colony forming units (CFUs) on the selective and non-selective plates. The competitions were performed in triplicates.

### Western blotting

For sample collection, killing buffer is added, followed by a centrifugation at 4°C, 5000xg, 7 min. After two 1X PBS wash steps, the pellets are solubilized and sonicated (3×30 cycle, 1 s cycles, 80% Amplitude). Protein determination was performed using BCA protein assay kit from Pierce^TM^. Fifteen microgram of protein was loaded per lane and ran on 8–20% gradient polyacrylamide sodium dodecyl sulphate gels together with the ColorPlus prestained protein marker (NEB). The separated proteins were transferred on a 0.2 μm nitrocellulose membrane via semy-dry blotting. A list of antibodies and their dilutions is provided in Supplementary Table S1. ECF substrate for AP was used for protein detection. Protein quantification and image processing were performed on AIDA software, with subsequent evaluations in Microsoft Excel. All detections were normalized to the β’ subunit of RNA polymerase. In order to allow comparisons between separate experiments, the highest ratio was set as 100% for each blot and used to calculate the rest of the values in percentage. For multiple detections, the membranes were stripped with RotiFree stripping buffer at 50°C.

### High-resolution agarose gel-electrophoresis

pUC18 plasmid extraction was performed from WTeng, TL and TR strains at an hourly rate of the strain's growth with the Roti Prep Plasmid Mini kit. Five hundred nanogram of DNA together with a lambda PstI marker is loaded onto a 1% agarose, 1X TBE (Tris, Boric Acid, EDTA) gel with 1.5 μg/ml chloroquine and run for 23 h at 45V. The gels were stained with ethidium bromide. 1D evaluation scans were done with AIDA software and the intensity of the bands was plotted over their position in pixels. The starting point was selected from the well for each lane.

### Quantitative RT-PCR

Five nanogram of total RNA from a 1 ng/μl dilution was used per reaction in a total volume of 25 μl. RNEasy kit was used for RNA extraction and the QuantiTect SYBR® Green RT-PCR kit from Qiagen® was used for RT-PCR reactions. cDNA synthesis was carried out at 50°C for 30 min, followed by inactivation of reverse transcriptase and activation of DNA polymerase step at 95°C for 15 min. Amplification reactions consisted of 40 cycles with denaturation at 95°C for 15 s, primer annealing for 30 s (Supplementary Table S2) and primer extension at 72°C for 30 s. Fluorescence was recorded by the Stratagene MX3000P. Data analysis was done with LinRegPCR software (Version 2014.1) and Microsoft Excel. In all RT-PCR data representations the y-axis is ln*R*, where

\begin{equation*}_{\text{ }}^{R = {{{\rm Eff}^{Ct} \;{\rm Reference}}}}\left/ ^{\text{ }}_{{{\rm Eff}^{Ct}} \;{\rm Target}} \right.\end{equation*}

WTeng data was used as reference. Since a natural logarithm of the ratio is plotted, a value of 1 indicates a 2-fold increase, while a value of −1 indicates a 2-fold decrease. For the determination of gene copy numbers the total cellular DNA was isolated by a modified procedure using the Roti-Prep Plasmid kit (Roth) and the *fis* and *yfp* DNA was measured using specific primers (Supplementary Table S2) by qPCR.

### Oxidative stress assay

Overnight cultures from CSH50 wild-type strain, TL and TR were diluted 1:200 in fresh LB medium. The strains were grown in a Tecan M100 Infinite Pro, at 37°C with linear shaking, second amplitude (∼600 rpm). OD580 was recorded every 10 min. H2O2 was added to 5.5 mM f.c. to each growing culture at the beginning of exponential phase (around 60 min) or to a f.c. of 10 mM at the 8 h of growth (stationary phase). The survivors were quantitated at intervals as indicated.

### Minimal inhibitory concentration test

Bacterial suspension with a concentration of ∼10^6^ bacteria/ml was inoculated in microtiter plates. Antibiotic solutions were distributed from the first (A) row, in serial dilutions manner until one to the last (G) row. The last raw (H) contained no antibiotics and served as a control of uninhibited growth. Plates were incubated at 37°C for 12–14 h, after which OD600 of the cultures was measured and recorded. All experiments were carried out in Müller Hinton Broth medium.

### Biolog phenotypic microarrays and computation of the similarity tree

Biolog experiment was performed in duplicate for each analysed strain (42) using PM11 to PM20 comprising 240 different compounds in increasing concentrations (four dilution steps) and providing 7.680 data points in total used for computation of the similarity tree. In the first step of computation we investigated the distribution of growth values in all the analysed strains. The data showed no deviation in the distribution of growth values indicating no general growth deficiency of any strain and allowing an unbiased comparison of the samples. However, a pairwise t-test revealed significant differences between the strains in certain media. Based on the pairwise growth differences, we computed the Euclidean distance matrix of all pairs of samples. To obtain the similarity tree, we applied the Neighbor-Joining algorithm to the distance matrix.

## RESULTS

**Figure 1.**
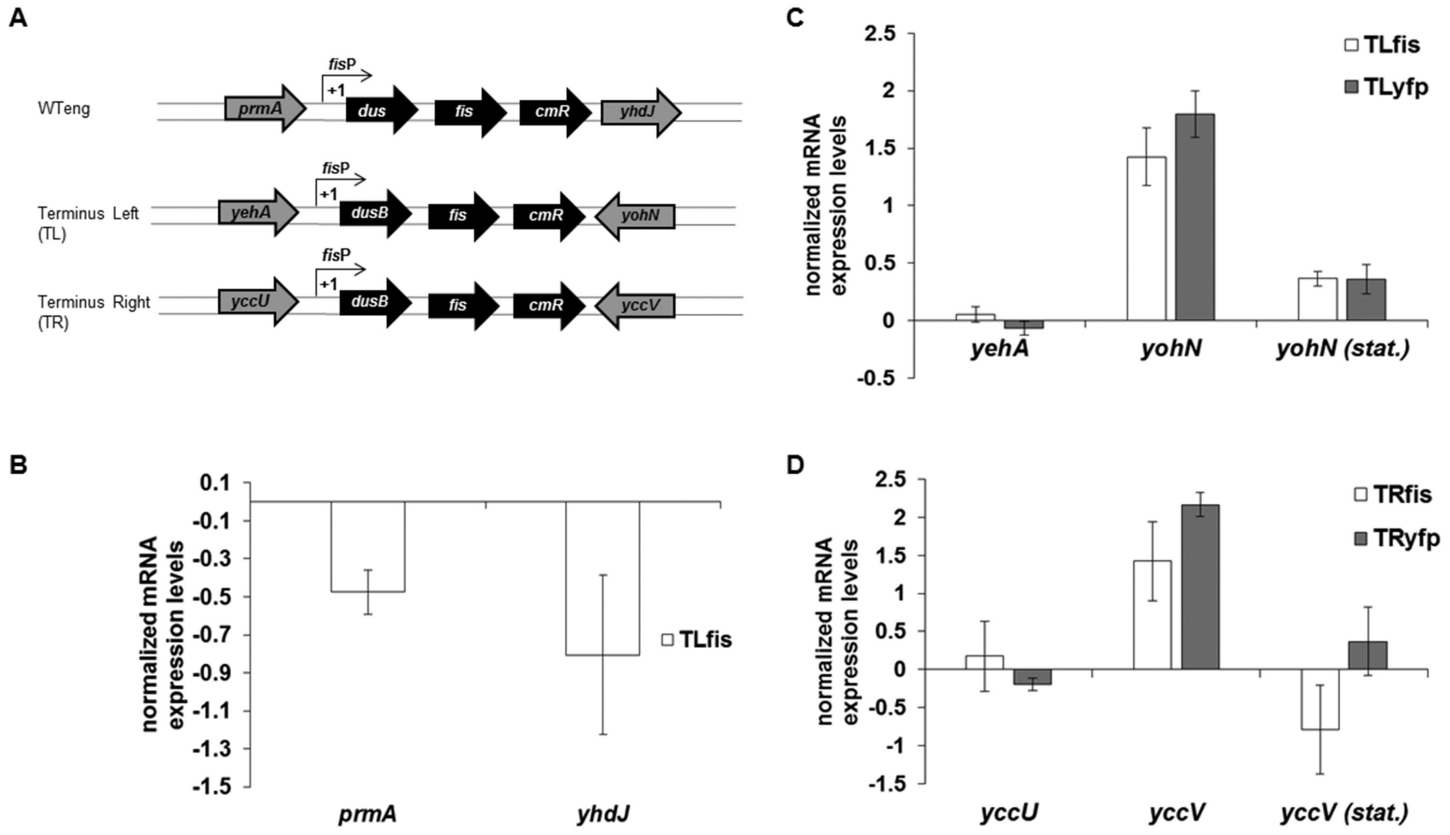
The effect of terminus-proximal *dusB-fis* insertions on flanking genes. (**A**) Schematic representation of the insertion construct *fisP-dusB-fis-cm* in its native locus (WTeng), in position TL, and TR along with the respective flanking genes. (**B**) Quantitation (qRT-PCR) of the mRNA levels detected for the genes flanking the original *fis* locus, TL (**C**) and TR (**D**) insertions. WTeng serves as a baseline. The Y-axis is on a logarithmic (ln) scale, such that a value of +1.0 and −1.0 respectively indicate a 2-fold increase and decrease compared to the baseline. Note that there are no substantial changes in the original locus after *fis* deletion. For both TL and TR, the upstream genes show no difference in expression to WTeng, whereas the downstream genes are upregulated, regardless of whether the insertion carries the *fis* ORF (TLfis/TRfis) or *yfp* ORF (TLyfp/TRyfp). This effect was observed only during exponential phase of growth and was absent in stationary phase (stat).

The abundant NAP FIS is a global regulator of cellular metabolism, coordinating chromosomal DNA topology and ribosomal biosynthesis with the growth conditions (28–32). The *dusB-fis* operon is located on the left chromosomal arm in the vicinity of OriC (Supplementary Figure S1). To test whether the chromosomal position of a regulatory gene can affect the cellular phenotype, the *dusB-fis* operon tagged with a chloramphenicol resistance cassette (*dusB-fis-cat*) was inserted between the convergent transcription units (in order to avoid interference with gene promoters and any upstream regulatory elements) in two locations in the vicinity of replication terminus on both arms of the chromosome (between *yehA* and *yohN* on the left, and *yccU* and *yccV* on the right replichore; Figure 1A; see also Supplementary Figure S1) of *E. coli* CSH50 Δ*fis* strain (Supplementary Table S3). To have an appropriate control for the strains carrying the moved *dusB-fis* operons (denoted hereafter as ‘movants’), the *dusB-fis-cat* construct was inserted also in the native locus (denoted hereafter as WTeng). Deletion of the *dusB-fis* operon did not show significant changes in the expression of *prm* and *yhdJ* genes flanking the original locus when compared to WTeng (Figure 1B). However, when we tested the effect of the moved *dusB-fis* operons on the local environment we found that both insertions increased the transcription of downstream genes, *yohN* and *yccV* (Figure 1C and D). This effect was observed in exponential phase and was absent in stationary phase, consistent with strong expression of *fis* during exponential phase (26). To distinguish whether this effect was due to the insertion as such, or to the expression of the *fis* gene message, we substituted the *fis* ORF in the *dusB-fis-cat* construct by a *yfp* ORF (*dusB-**yfp**-cat*) and observed a similar effect on downstream genes (Figure 1C and D). We infer that increased expression of the downstream genes during exponential phase is due to insertions as such and not to the expression of the *fis* gene message.

### The *fis* chromosomal position shift affects the competitive growth fitness

**Figure 2.**
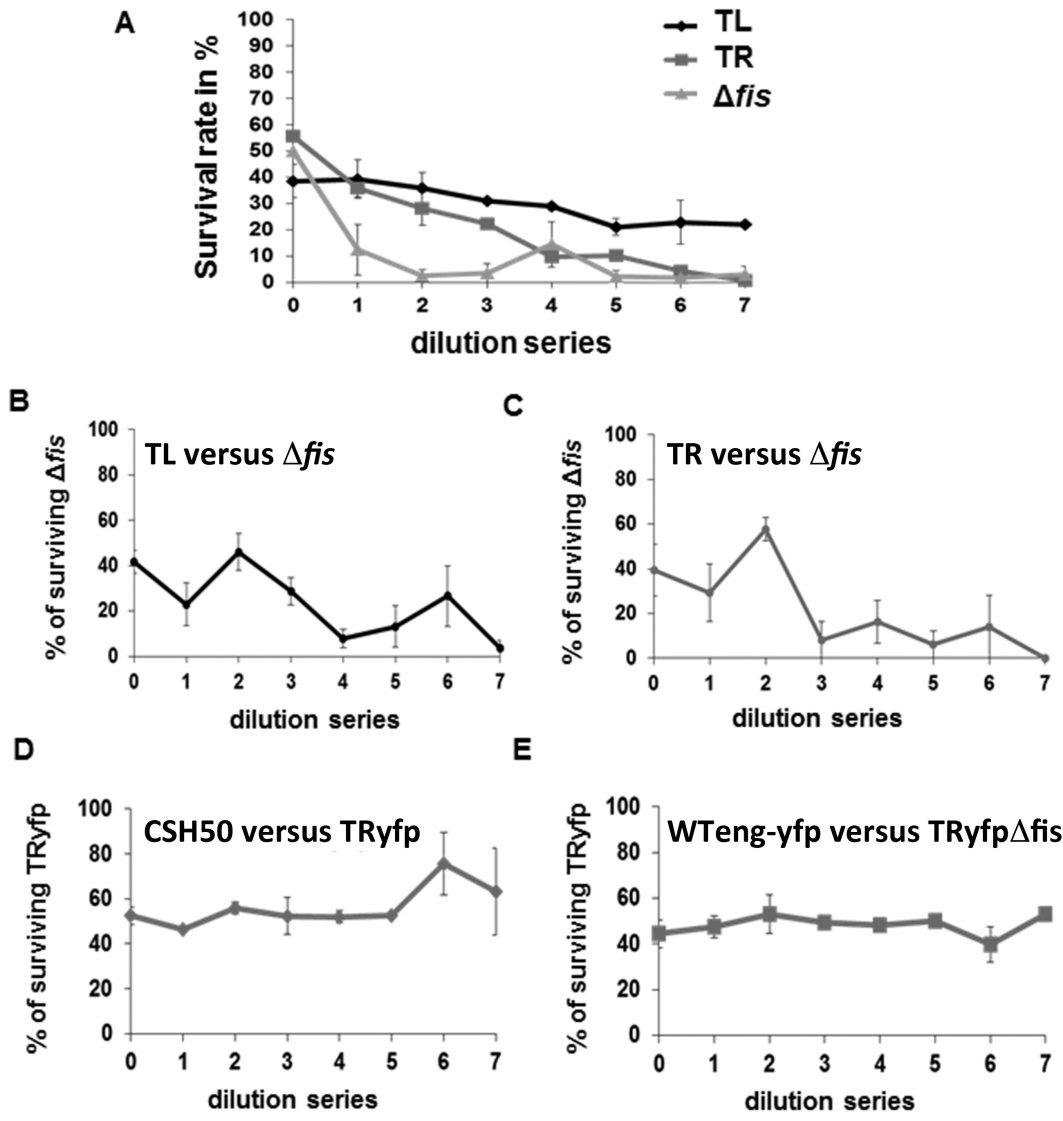
Competitive growth fitness assay. (**A–E**) For all competition assays, the cells were mixed in a 1:1 ratio. After 12 h of growth two equal aliquots were plated on selective plates and non-selective solid medium and the survival ratios calculated by counting of the CFUs for both strains in the mixture. The mixed cultures were again diluted 1:10 000 in fresh medium and allowed to grow for further 12 h. The procedure was repeated seven times. Abscissa—number of dilution/plating cycles; ordinate—percentage of survivors. (A) The growing WTeng cells have selective advantage over the TL, TR and Δ*fis* cells. (B, C) Competitive fitness assays of TL and TR versus *Δfis* demonstrate that both movants have a selective advantage over the mutant. (D, E) Control competitive fitness assays of TRyfp carrying the *yfp* ORF instead of the *fis* ORF mixed with either the parent CSH50 strain (D) or with WTeng strain carrying the *yfp* ORF instead of the *fis* ORF in the native *fis* locus (E). Since WTeng-yfp is lacking *fis*, in this latter experiment the TRyfp Δ*fis* strain was used for the competition. All error bars are standard errors.

We next tested the effect of the insertions on the competitive growth fitness. Separately, the movants grew similarly, being indistinguishable in all the tested growth media from either the strain carrying the *dusB-fis-cat* construct in the original position (WTeng) or the CSH50 parent strain (Supplementary Figure S2). For growth competition the overnight cultures of the movant and WTeng strains were mixed in equal proportions before inoculation in rich medium. The growing co-cultures were re-inoculated after every 12 h and the cell composition monitored by counting the CFUs on agar plates. Both movants showed reduced fitness compared to WTeng, albeit to different extents. The CFUs for the movant strain with terminus-proximal insertion in the left chromosomal arm (TL) dropped to 20% after the fifth dilution and remained at this level until the end of the experiment, whereas the strain with terminus-proximal insertion in the right arm (TR) was competed out after the sixth dilution (Figure 2A). At the same time, both movants competed out the *fis* mutant, although in this respect they were less efficient than WTeng (compare Figure 2A, B and C). Importantly, the TRyfp insertion strain carrying *dusB-**yfp**-cat* instead of *dusB*-***fis****-cat* did not show any reduction in competitive fitness when compared to the original CSH50 wild-type strain (Figure 2D). Also the WTeng strain carrying *dusB-**yfp**-cat* instead of *dusB*-***fis****-cat* in the original locus (WTeng-*yfp*; note that this substitution renders the strain a *fis* mutant) did not show any growth advantage over the TR*yfp*Δ*fis* strain (Figure 2E). Thus although the *dusB–**yfp*** insertions in the Ter region, alike the *dusB-**fis*** insertions affect the expression of downstream genes, they do not compromise competitive growth fitness (Figure 2D and E) and therefore, altered expression of downstream genes cannot explain the impaired competitive fitness of the movants. Note also that both the Ter-proximal *dusB-**yfp**-cat* and *dusB*-***fis****-cat* insertion constructs reposition the *dusB* gene in the chromosome. This indicates that repositioning of *dusB* (see Figure 1) cannot account for the reduced competitive fitness of the movants. Therefore, we attribute the latter effect primarily to chromosomal repositioning of the *fis* gene.

### The *dusB*-*fis* chromosomal position shift affects *fis* expression

**Figure 3.**
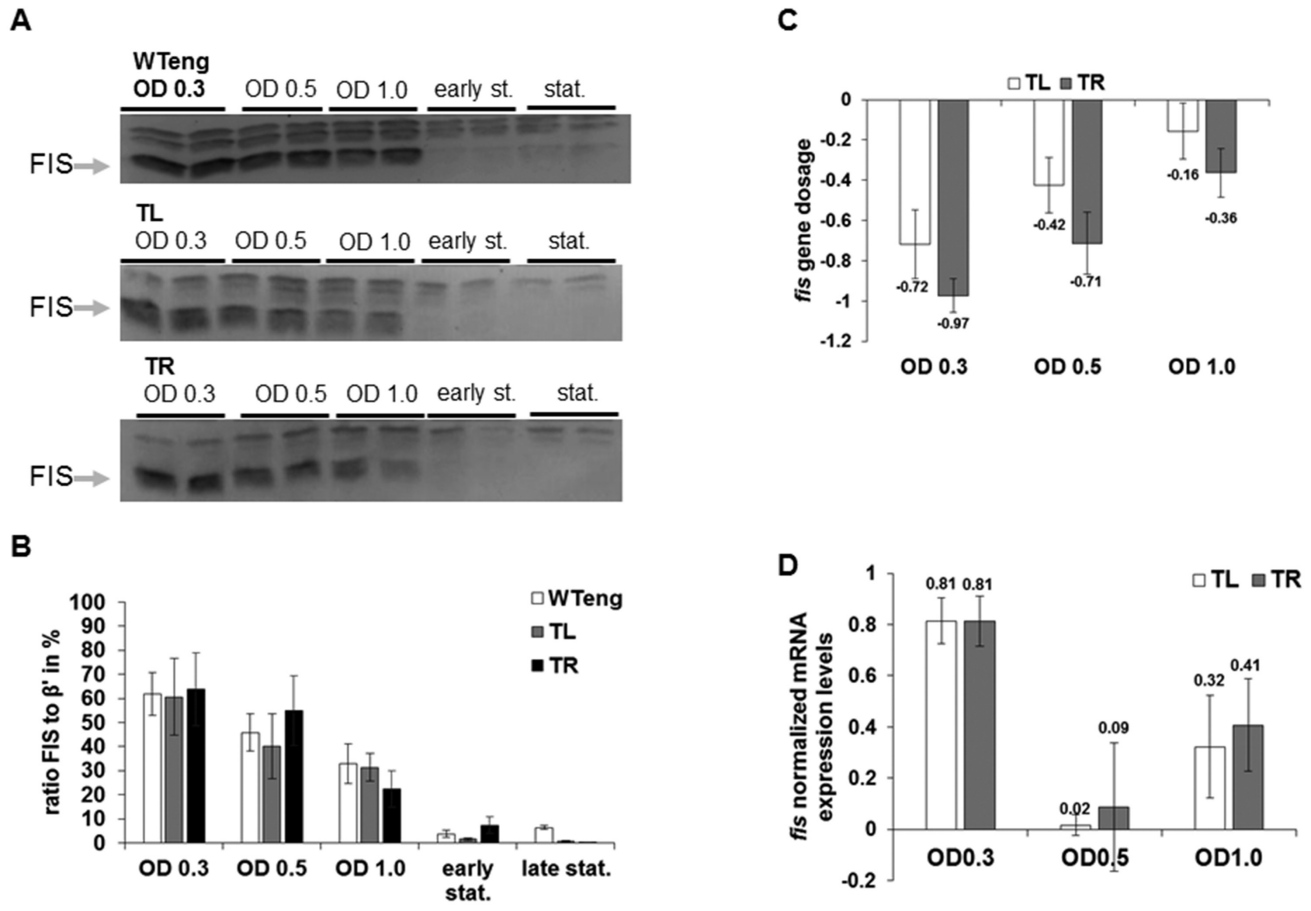
The unaltered expression of *fis* in the movant strains is compensated. (**A, B**) Western blots showing FIS protein expression in WTeng, TL and TR strains (A) and the respective quantification (B), normalized to the β’ subunit of RNA polymerase. All quantifications were performed with AIDA software. In order to compare the western blots, the ratios to β’ were converted into percentages. (**C**) Determination of *fis* gene dosage in TL and TR strains during exponential growth phase. The values determined for WTeng serve as a baseline. The Y-axis represents the *fis* gene dosage in logarithmic (ln) scale, such that a value of −1.0 entails a 2-fold decrease compared to the baseline. (**D**) *fis* mRNA expression levels recorded during exponential phase with WTeng serving as a baseline. The Y-axis represents *fis* expression in logarithmic (ln) scale, therefore a value of +1.0 means a 2-fold increase compared to the baseline. All error bars are standard errors.

The expression of *fis* demonstrates a remarkable transient pattern with sharp increase on the entry of cells in exponential phase and subsequent rapid decrease (26). To test whether the positional shift of the *dusB-fis* operon affects the expression of the *fis* gene we monitored the pattern of FIS expression in WTeng and both the TL and TR movants. The strains were inoculated in fresh medium and the amount of FIS was detected by western blot analyses in crude extracts of cells harvested at intervals after inoculation at the same optical density. A typical pattern of FIS expression was observed in all strains, and the amount of FIS protein in the extracts was also similar (Figure 3A and B). However, we detected a roughly 2-fold decrease of the *fis* gene copy numbers in the movants, consistent with the location of the TL and TR insertions in the vicinity of chromosomal replication terminus (Figure 3C). To explore how the fewer gene copies in movant strains could produce amounts of FIS protein similar to wild type, we compared the *fis* gene transcription in WTeng, TR and TL strains. We observed that the transcription of *fis* was increased roughly twice in the movants compared to WTeng at OD 0.3 (Figure 3D). This increased transcription was evident also at OD 0.5, where despite the still lower gene dosage in the movants the detected *fis* mRNA levels were similar to those of the wild-type control.

To distinguish whether this increased expression was due to repositioning of the *fis* promoter or the *fis* gene, we tested the expression of the *dusB-**yfp**-cat* constructs in a *fis* mutant strain. The constructs were inserted once in the same terminus-proximal position on the left arm (TL), and once in the middle of the left arm (ML). Compared to the *dusB-**yfp**-cat* insertion in the native locus, the repositioned *dusB-**yfp**-cat* constructs showed similar reduction in copy number as observed for *dusB*-***fis****-cat*, yet no enhancement of transcriptional activity was observed (Supplementary Figure S3). This indicates that the presence of the *fis* ORF in the construct is responsible for the increased transcription observed in the movants. We thus infer that the chromosomal position shift of the *dusB-fis* operon towards the replication terminus results in its increased transcription. This increased transcriptional activity of the repositioned operons, compensating for the decreased *fis* gene copy number, explains the similar FIS levels in the WTeng and movant strains.

### The *dusB-fis* chromosomal position shift modulates the DNA supercoil dynamics

**Figure 4.**
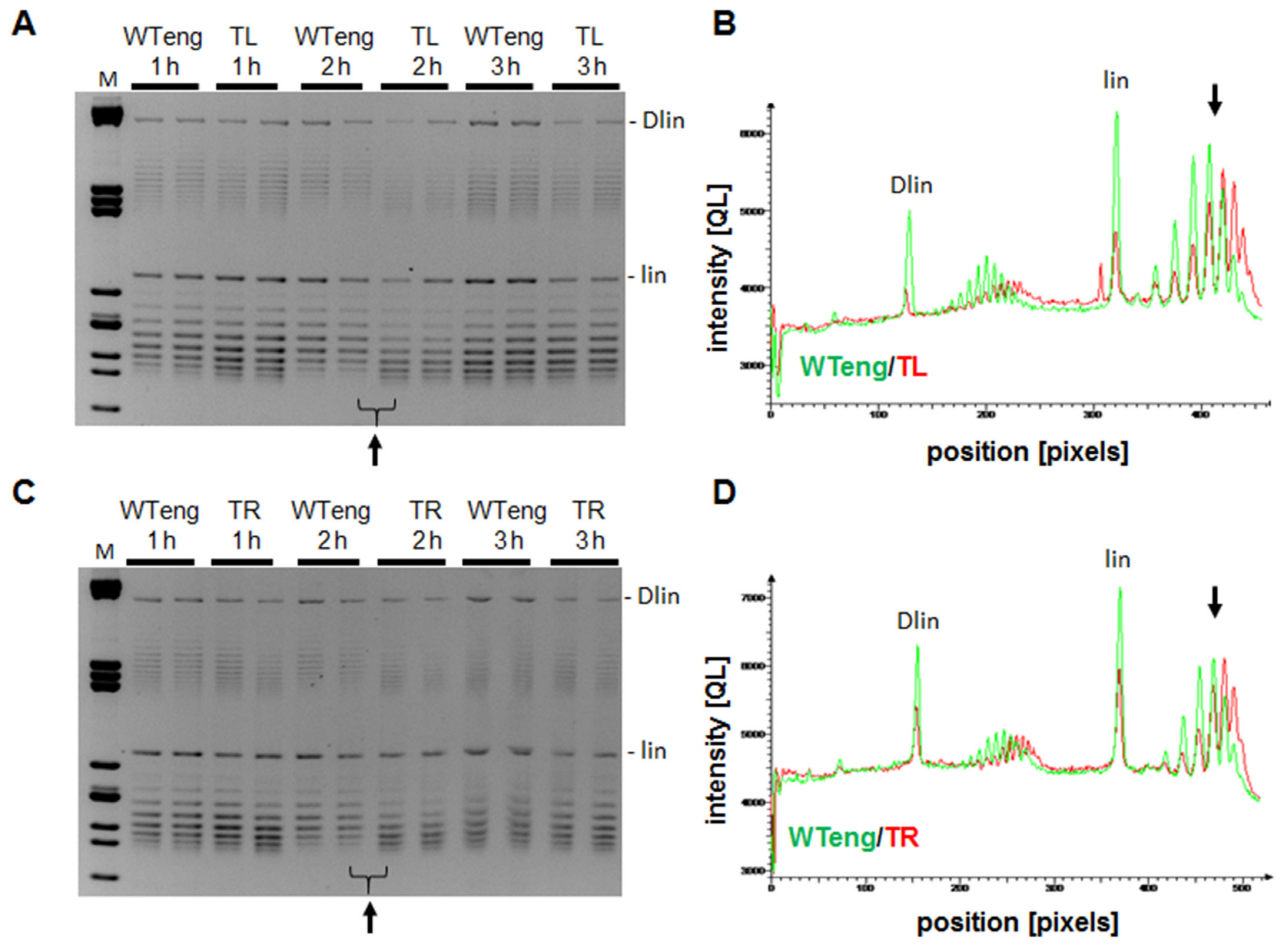
The TR and TL movant strains show an altered distribution of plasmid topoisomers during the growth cycle. (**A**) High-resolution agarose gel-electrophoresis of pUC18 plasmids isolated from WTeng and TL, grown in rich medium. (**B**) 1D scan of the tracks indicated by a bracket in panel (A), performed with AIDA software. Green and red colours indicate the distribution of topoisomers in respectively WTeng and TL at 2 h after inoculation. The intensity of the bands is plotted against their position on the gel, with 0 pixels indicating the edge of the wells. A clear shift in the distribution of the plasmid populations is recorded both for the dimeric and monomeric species. Note that the reference point (migration) of the linear dimeric and monomeric plasmids (dlin and lin, respectively) is unaltered. (**C, D**) Same as (A) and (B), but for plasmids isolated from WTeng and TR. Gel-electrophoresis was carried out in the presence of 1.5 μg/ml chloroquine.

Since FIS is involved in homeostatic regulation of the chromosomal DNA topology (31,33–34), we asked whether the positional shift of the *dusB-fis* operon affects the global topology of the DNA. For this purpose we transformed pUC18 reporter plasmids in WTeng, TR and TL strains and investigated the distributions of plasmid topoisomers during the growth by high-resolution agarose gel-electrophoresis. We found that the growth phase-dependent distribution of the plasmid topoisomers was noticeably affected in both the TR and TL strains. In particular, the reporter plasmids consistently showed an increased level of supercoiling in both movants compared to WTeng (/ΔLk/ ≥1), being most conspicuous at 2 h after inoculation (Figure 4). Thus, despite similar intracellular FIS concentrations, the growth phase-dependent dynamics of plasmid DNA supercoiling in the movants and wild-type cells differ.

### The *dusB-fis* chromosomal position shift modulates the overarching network

FIS is a component of an overarching network of the NAPs, DNA topoisomerases and components of transcription machinery regulating the growth phase-dependent changes of DNA topology in *E. coli* (7). Therefore, we asked whether the positional shift of *dusB*-*fis* operon could affect the state of this network. For this purpose, we measured the protein levels of the network components in growing WTeng and movant strains by western blot analyses. Overall, the changes detected in both movants were remarkably similar (Table 1). Although moderate (15–25%) deviations in the protein levels were observed for many of the analysed network components (Supplementary Figure S4), substantial differences were observed for the highly abundant NAPs, H-NS (1.76-fold and 2.1-fold increases respectively in TL and TR during late stationary phase) and Dps (1.51-fold and 1.74-fold increases during transition, and 1.54-fold and 1.44-fold increases during late stationary phase in TL and TR respectively), as well as the RNA polymerase (RNAP) sigma initiation factor for flagella biosynthesis RpoF (1.50-fold and 1.91-fold increases during early stationary phase in TL and TR, respectively). The A subunit of DNA gyrase (GyrA) was slightly elevated (1.29-fold) in the TR movant on transition to stationary phase. We verified the observed deviations in GyrA protein levels by qRT-PCR analyses and found that in TR, consistent with elevated protein levels in this movant, the amount of the *gyrA* transcript was significantly increased, whereas we could not detect any significant alterations of topoisomerase I (*topA*) expression (Supplementary Figure S5). We infer that positional shift of the *dusB-fis* operon affects the state of the overarching network leading to conspicuous changes in protein levels of several network components.

**Table 1.** Fold changes in protein expression for members of the overarching network, normalized to WTeng

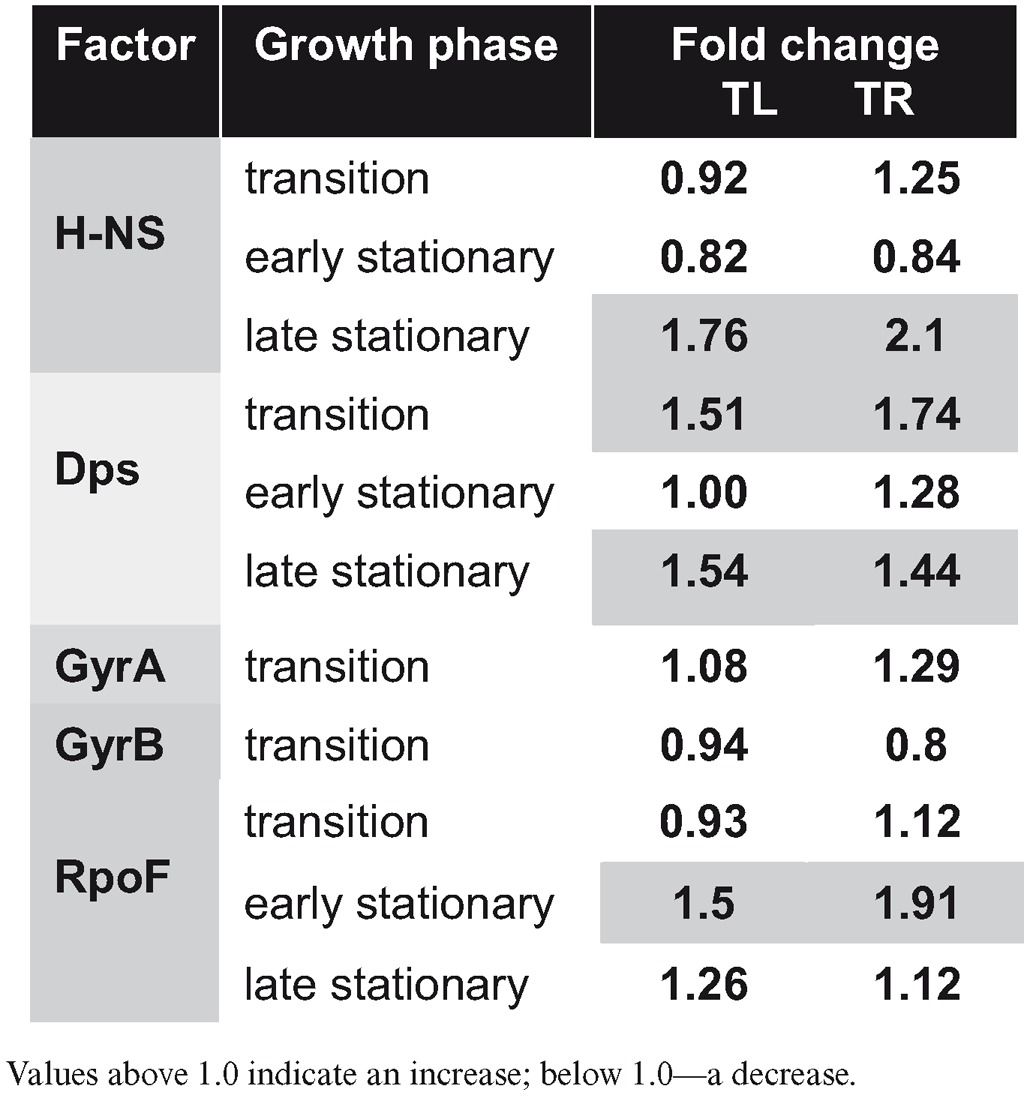

### The *dusB-fis* chromosomal position shift modulates the response to oxidative stress

**Figure 5.**
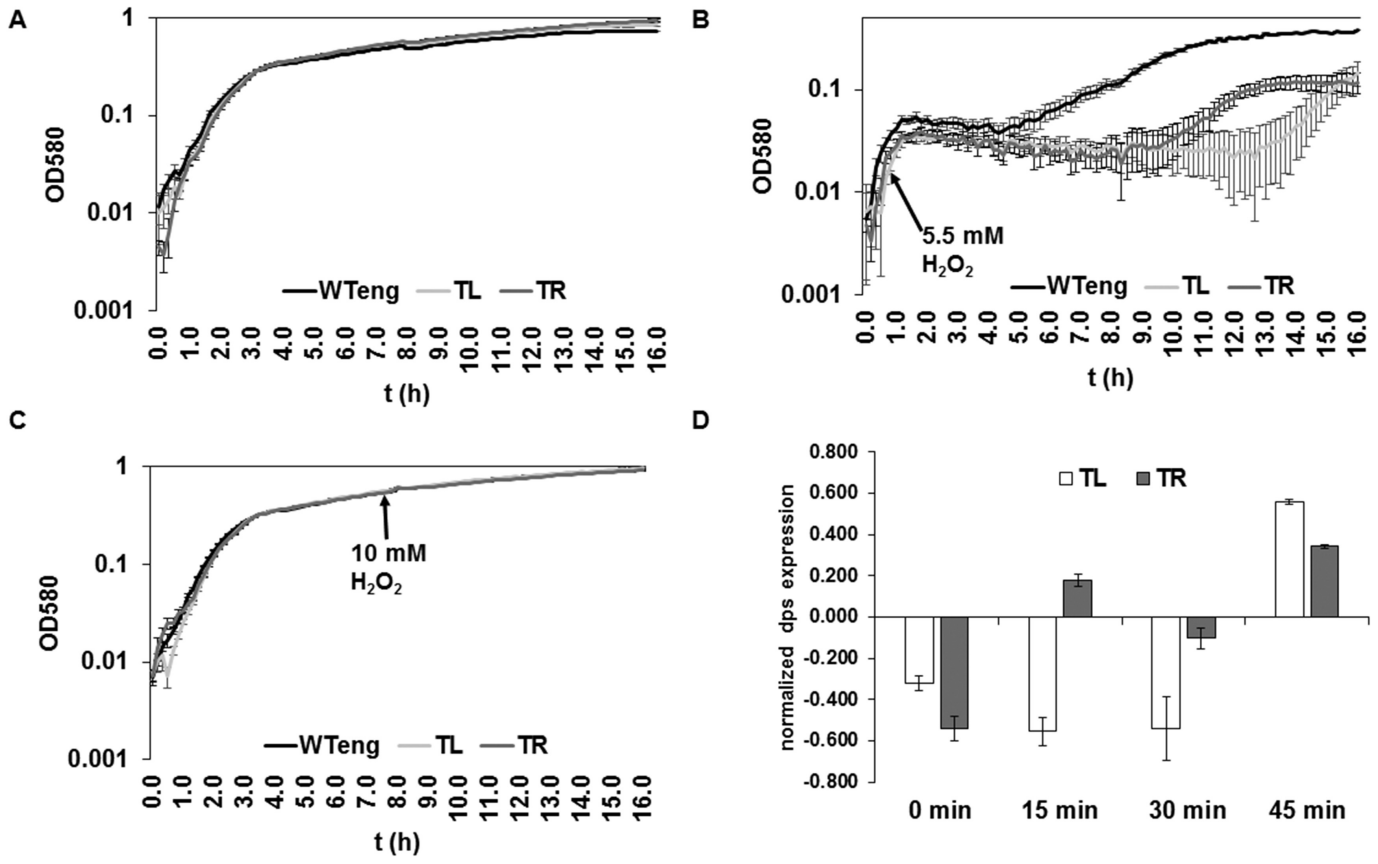
Response of the TR and TL movants to oxidative stress. (**A–C**) Peroxide shock treatment. (A) Growth curves of WTeng, TL and TR over 16 h without any treatment. (B) 5.5 mM H2O2 were applied on the onset of exponential phase to each culture (indicated by arrow), and the subsequent growth was monitored over 16 h. TL and TR (light and dark grey lines) show impaired ability to cope with the peroxide shock, unlike WTeng (black line). (C) 10 mM H2O2 were applied during the 8 h of growth (stationary phase) to each culture (indicated by arrow), and the subsequent growth was monitored over 16 h. In stationary phase there is no observable difference in the growth. (**D**) *dps* mRNA expression levels before and after peroxide treatment (6 mM) with WTeng serving as baseline. The Y-axis represents the normalized expression in logarithmic (ln) scale with a value of +1.0 indicating a 2-fold increase. The negative values indicate correspondingly a decrease compared to the baseline. All error bars are standard errors.

The overarching network of global regulators mediates the adaptation of the cells to changing environment (7). Exposure of exponentially growing cells to oxidative stress induces production of Dps protecting the cells from oxidative stress, whereas FIS acts as a repressor of the *dps* gene during exponential growth (35,36). Since the levels of Dps were found altered in both movants, we compared the response of the WTeng and movant strains to oxidative stress applied both in exponential and stationary phase. We found that during exponential growth both movants were more sensitive to oxidative stress than wild type, TL being affected more severely (Figure 5A and B). Accordingly, when we measured the *dps* transcription in the movants during exponential growth we found lower mRNA levels associated with temporal alteration of the *dps* transcriptional response to oxidative stress and again, TL showed a stronger deviation (Figure 5D). However, when oxidative stress was applied to cells in stationary phase, no differences were observed as expected, since at this growth stage the movants demonstrate elevated levels of Dps compared to wild type (see Table 1). Taken together, these findings suggest that the chromosomal position shift of the *dusB-fis* operon deregulates *dps* expression and thus impairs the adaptive response of the exponentially growing movant strains to oxidative stress.

### The *fis* chromosomal position shift modulates the sensitivity to drugs

**Figure 6.**
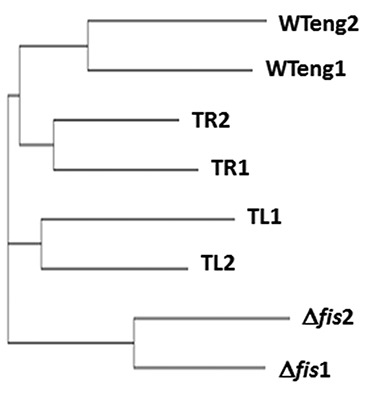
Similarity tree computed from the response of the movants to 240 various antibiotics and chemicals in the PM (Biolog) screening experiment. The similarity tree shows that in terms of the global response the TL and TR movant strains are situated between WTeng and Δ*fis*, with TL being closer to Δ*fis* and TR to WTeng.

We explored the pleiotropic effect of the positional shift of *dusB-fis* operon by testing the sensitivity of strains to different groups of hazardous substances using Phenotypic Microarray (PM) analyses (37) comprising 240 different compounds including antibiotics, DNA intercalating agents, oxidizing compounds, detergents and toxic anions. In these experiments we compared the sensitivity patterns of TL and TR movants to both, the WTeng and *fis* mutant strains. Among these four strains, we detected strong differential responses to 27 compounds representing all the different groups. The WTeng and both movant strains were found to be more resistant to fluoroquinolone gyrase inhibitors than the *fis* mutant. At the same time only the WTeng strain, but not the TR and TL movants, differed from *fis* mutant regarding the sensitivity to a number of compounds including oxidizing agents, toxic anions and cations, and intercalator drugs (Table 2). Computation of the similarity tree based on the sensitivity patterns of the four strains to all 240 tested compounds showed that the susceptibility phenotypes of the movants were intermediate between WTeng and the *fis* mutant, with TR being closer to WTeng and TL closer to the *fis* mutant (Figure 6). Our results are consistent with earlier observations of altered drug tolerance in the *fis* mutant strain (38) and demonstrate that the chromosomal positional shift of the *dusB-fis* operon modulates the response to toxic compounds and the antibiotic susceptibility of *E. coli* cells.

**Table 2.** Antibiotic and chemical sensitivity of the strains

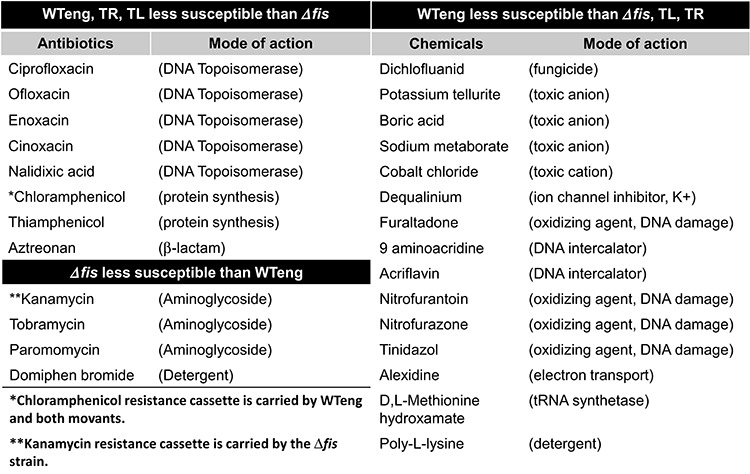

## DISCUSSION

In this study we set out to test the prediction that a change in the chromosomal position of a global regulatory gene could alter the bacterial phenotype. To do this we moved the *dusB-fis* operon of *E. coli* from its original position in the vicinity of OriC, to two new positions equidistant from *OriC*, but close to the replication terminus on opposite arms of the chromosome. The repositioned operon contained the promoter upstream sequences comprising all the known binding sites for regulatory proteins involved in *fis* transcriptional control (39–41). We used the entire operon instead of the *fis* gene, because the upstream *dusB* ORF is absolutely required for efficient translation of the *fis* mRNA, as well as substituted reporter gene message (42–44). We observed that although the displaced operons quantitatively maintained the growth phase-dependent expression pattern and produced the same amount of FIS protein in the cell, the repositioning of the *dusB-fis* operon exerted pleiotropic effects on the expression of genes distant from the insertion site.

We found that terminus-proximal insertions increased the transcription of downstream genes during the exponential, but not stationary phase, suggesting that the increased activity of the *dusB-fis* operon during exponential growth affects the downstream genes. A similar effect was reported in a recent study made with *lac* promoter-reporter fusion cassette, but in that case chromosomal insertions repressed downstream gene transcription (25). The reporter cassette used in the cited study (i) carried a truncated version of the *lac* promoter inducible by IPTG, whereas *fisP* is a growth phase- and rate-dependent promoter; (ii) was substantially smaller in size, comprising ∼200 bp of the *lac* promoter regulatory sequence plus the *gfp* gene, whereas in our case the entire *dusB-fis* operon comprised over 1.4 kb plus the 900 bp chloramphenicol resistance cassette, such that the operon was separated from downstream genes by the resistance cassette; (iii) carried an upstream, but not a downstream, transcriptional terminator, whereas in our construct the *dusB*-*fis* locus was insulated by natural terminators flanking the operon. Unlike the inducible *lac* promoter, *fis* promoter activity is exquisitely sensitive to changes of DNA supercoiling, due in part to the GC-rich discriminator sequence between the −10 hexamer and the transcription start site (45), which mediates the growth phase- and growth rate-dependent control (26,28). This sensitivity to supercoiling might also be related to the observations that the *fis* promoter region, highly conserved in all pathogenic *E. coli* and *Shigella* strains, is characterized by strong intrinsic curvature (46), is regulated by a higher-order RNA polymerase complex involved in sensing the topological state of DNA (41,44) and appears evolutionarily streamlined to act as a topological switch (47,48). Some or all of these differences could contribute to disparate effects exerted by the insertions of *lac* and *fis* promoter constructs on their immediate surroundings. However, the changes in transcription of the neighbour genes caused by *dusB-fis* operon insertions cannot explain the observed phenotypic changes. Whereas we find that chromosomal displacement of the *dusB-fis* operon affects the competitive growth fitness (Figure 2), we can rule out the role of activation of downstream genes in this effect, because substitution of *yfp* for *fis* in the insertion construct similarly increases downstream transcription without impairing competitive fitness. The same is true for the other phenotypic changes observed in the movants (data not shown). Therefore, we attribute the observed pleiotropic effects of the displaced operons primarily to the chromosomal position shift of the *fis* gene.

In fast growing *E. coli* cells the time needed for replication of the chromosome exceeds the time needed for division, and therefore new rounds of replication start before the earlier forks have reached the terminus, resulting in a higher gene copy number in the vicinity of the replication origin compared to the terminus (49). The important observation is that despite the decreased *fis* gene copy number due to the Ter-proximal location in the movants, the intracellular concentration of FIS is similar to wild type at the expense of increased transcription of repositioned *fis* gene (Figure 3). This finding suggests that the lowered *fis* gene dosage is compensated by increased transcription. A similar compensatory effect has been observed previously in the case of ribosomal operons regulated by FIS. It was shown that inactivation of several out of the seven ribosomal RNA operons in *E. coli* leads to an increased expression of the remaining operons (50). The ribosomal operons are subject to complex feedback regulation, but the actual control mechanism still remains controversial (review (51)). However, the activity of ribosomal operons was shown to correlate with copy number effects—that is, the operon was expressed more strongly when it was closer to the origin (52). In contrast, with the *dusB-fis* operon subject to autoregulation by FIS we find the opposite—the terminus proximal copies are expressed more strongly. Furthermore, we observe similar pleiotropic effects induced by *dusB-fis* operons located in dissimilar local environments of the left and right chromosomal arms, yet at similar distances from the chromosomal end. This suggests a mechanism sensing the *fis* positional shift along the OriC-Ter axis and homeostatically adjusting the *fis* transcription strength. The crucial role of FIS autoregulation in this adjustment is indicated by experiments on *fis* mutant strains carrying terminus-proximal *dusB-**yfp**-cat* constructs, in which no such adjustment of *fis* transcription strength is observed (Supplementary Figure S3). By the same token, we can rule out that the increased activity of the terminus-proximal *dusB-fis* operons reflects an enhanced accessibility to the transcriptional activator IHF encoded by the terminus-proximal *ihfA* and *ihfB* genes (39,40). Therefore, we prefer a simple explanation for increased *fis* expression based on recently reported dependence of the diffusion of transcription factors on their production site (24). Since *fis* expression is subject to negative autoregulation (27), and since in contrast to the native locus relatively rich in FIS binding sites, both the TR and TL insertions reside in chromosomal regions relatively poor in FIS binding sites (see Supplementary Figure S1), we propose that *fis* autoregulation is modified by altered diffusion of the FIS protein from its production site in different (OriC- and Ter-proximal) chromosomal environments. Furthermore, in growing cells the supercoiling level of the chromosomal OriC- and Ter-proximal regions may be different, whereas binding of FIS is shown to depend on DNA topology (6,33). This hypothesis can be tested by insertions of extended arrays of high-affinity FIS binding sites at different distances from the *fis* gene and comparisons of *fis* expression and binding site occupation at different supercoiling levels, but testing this certainly merits a separate study.

Overall, in both the TL and TR strains we observed very similar phenotypic effects regarding the changes in competitive fitness (Figure 2), growth phase-dependent dynamics of DNA topology (Figure 4), protein levels of the overarching network components (Table 1), sensitivity to stress (Figure 5) and to hazardous substances including antibiotics (Table 2). These similarities support the notion that the effects of regulatory genes depend on their position along the OriC-Ter axis of the bacterial chromosome (6). Nevertheless, although directionally similar, the effects were not identical and we observed also conspicuous differences. Firstly, the competitive growth fitness of the movants was impaired to different extents. Secondly, whereas we found similar changes of plasmid topology in both movants, the expression of the *gyrA* gene, which is regulated by FIS (30), and the corresponding GyrA protein levels (Table 1; Supplementary Figure S5) were significantly elevated only in the TR strain. Thirdly, in both movants we find lower transcript levels of the *dps* gene, which is repressed by FIS during exponential growth (35), and elevated levels of the Dps protein in late stationary phase compared to wild type (Figure 5D; Table 1). Accordingly, both movants are more susceptible to oxidative stress applied during early exponential but not stationary phase (Figure 5B and C). Nevertheless, the *dps* expression shows a different response to oxidative stress in TR and TL strains, being more similar to wild type during the first 30 min in the former (Figure 5D). Finally, the level of RpoF (FliA) sigma factor involved in regulation of flagella biosynthesis and motility was elevated during early stationary phase in both movants (Table 1), yet only TR showed an altered swarming capacity (data not shown). Given the similar levels of FIS in all studied strains (Figure 3A and B), this indicates that the strategy of chromosomal position shifts of regulatory genes is capable of unveiling deviations from the established control mechanisms and is thus instrumental in extending our understanding of genetic regulation by including positional information.

A striking observation of this study is that positional shift of the *fis* gene, very much alike the *fis* gene deletion, exerts pleiotropic effects in an otherwise wild-type genetic background. For example, in terms of global sensitivity patterns to 240 different tested compounds detected by PMs the movants are situated between the wild type and the *fis* mutant (Figure 6). Previous screening of the library of *E.coli* mutants for strains with altered antibiotic tolerance identified deletions in *fis* gene (38) reducing the level of multidrug-resistant ‘persister’ cells after treatment with gyrase inhibitor ofloxacin. In keeping with this we found that the wild type and movant strains are less susceptible to gyrase inhibitors than the *fis* mutant (Table 2). In particular, we found that the minimal inhibitory concentration for wild type and movant strains to gyrase inhibitors ciprofloxacin and norfloxacin was twice as high (0.25 μg/ml) as that for the *fis* mutant (0.125 μg /ml). However, despite being similar to wild type regarding the fluoroquinolone drugs, the movants were clearly different from wild type and more similar to *fis* mutant in terms of susceptibility to toxic anions and cations, intercalators and oxidizing agents (Table 2), the latter being consistent with their distinct response to oxidative stress (Figure 5B).

Our study is consistent with recent reports that chromosomal position shift can affect the reporter gene expression (25,53) but importantly, shows in addition that repositioning of a global regulatory gene, *fis*, substantially and stably alters the cellular phenotype regarding the competitive growth fitness, response to environmental stress and sensitivity to drugs. The phenotypic changes induced by the displacement of the *dusB-fis* operon occur under conditions of increased FIS production compensating for the lower *fis* gene copy numbers in the movants and leading to FIS levels indistinguishable from wild type and therefore, cannot be explained by alterations in FIS concentration, but perhaps by difference in availability of binding sites. The observed pleiotropic effect indicates that altering of the genomic coordinates of an important chromosome-shaping component can induce (very much like a gene mutation) a change in the state of the overarching regulatory network. Most conspicuous changes were observed in the levels of the abundant NAPs directly regulated by FIS - Dps and H-NS (Table 1), and these changes can have genome-wide effects, especially since FIS and H-NS directly compete for binding sites in the genomic promoter regions, and FIS also counteracts the Dps-dependent condensation of the genome (54). Furthermore, both Dps and H-NS are known to mediate the cellular response to environmental stress, whereas H-NS, alike FIS, is implicated in homeostatic control of supercoiling response (31), as well as in modulating the persister cell levels in response to antibiotics (38,55–56). All these parameters are modified in the movants and the similarity of phenotypic responses suggests that the crosstalk between the NAPs, and so the state of the overarching network, can be changed in a regular way by shifting a pleiotropic locus along the chromosomal OriC-Ter axis.

Notwithstanding the observation that in *E. coli* certain mRNA messages can be selectively targeted to particular subcompartments of the cell (57), our findings reveal the functional relevance of conserved gene order along the chromosomal OriC-Ter axis (6), underscore the critical role of gene position in the chromosome (23,24) and are consistent with the view that bacterial chromosomes respond to perturbations as integrated entities (58). More compellingly, the pleiotropic effect of gene position shift on cellular phenotype provides a new dimension for in-depth investigations of the organizational flexibility of transcriptional regulation and in addition, paves a way towards a novel experimental strategy of deliberately rewiring the genetic control by changing the chromosomal gene coordinates. We believe that application of this strategy will greatly facilitate the engineering of bacterial strains with useful properties for purposes of synthetic biology and biotechnology.

## Supplementary Material

SUPPLEMENTARY DATA

## References

[1] Wegner A.S., Alexeeva S., Odijk T., Woldringh C.L. (2012). Characterization of Escherichia coli nucleoids released by osmotic shock. J. Struct. Biol..

[2] Postow L., Hardy C.D., Arsuaga J., Cozzarelli N.R. (2004). Topological domain structure of the Escherichia coli chromosome. Genes Dev..

[3] Valens M., Penaud S., Rossignol M., Cornet F., Boccard F. (2004). Macrodomain organisation of the Escherichia coli chromosome. EMBO J..

[4] Yazdi N.H., Guet C.C., Johnson R.C., Marko J.F. (2012). Variation of the folding and dynamics of the Escherichia coli chromosome with growth conditions. Mol. Microbiol..

[5] Umbarger M.A., Toro E., Wright M.A., Porreca G.J., Bau D., Hong S.H., Fero M.J., Zhu L.J., Marti-Renom M.A., McADams H.H. (2011). The three-dimensional architecture of a bacterial genome and its alteration by genetic perturbation. Mol. Cell.

[6] Sobetzko P., Travers A., Muskhelishvili G. (2012). Gene order and chromosome dynamics coordinate gene expression during the bacterial growth cycle. Proc. Natl Acad. Sci. U.S.A..

[7] Travers A., Muskhelishvili G. (2005). DNA supercoiling – a global transcriptional regulator for enterobacterial growth. Nat. Rev. Microbiol..

[8] Browning D.F., Grainger D.C., Busby S.J. (2010). Effects of nucleoid-associated proteins on bacterial chromosome structure and gene expression. Curr. Opin. Microbiol..

[9] Dillon S.C., Dorman C.J. (2010). Bacterial nucleoid-associated proteins, nucleoid structure and gene expression. Nat. Rev. Microbiol..

[10] Rimsky S., Travers A. (2011). Pervasive regulation of nucleoid structure and function by nucleoid-associated proteins. Curr. Opin. Micro..

[11] Azam A.T., Iwata A., Nishimura A., Ueda S., Ishihama A. (1999). Growth phase-dependent variation in protein composition of the Escherichia coli nucleoid. J. Bacteriol..

[12] Murphy L.D., Zimmerman S.B. (1997). Isolation and characterization of spermidine nucleoids from Escherichia coli. J. Struct. Biol..

[13] Ohniwa R.L., Ushijima Y., Shinji S.S., Morikawa K. (2011). Proteomic analyses of nucleoid-associated proteins in Escherichia coli, Pseudomonas aeruginosa, Bacillus subtilis, and Staphylococcus aureus. PLoS ONE.

[14] Berger M., Farcas A., Geertz M., Zhelyazkova P., Brix K., Travers A., Muskhelishvili G. (2010). Coordination of genomic structure and function by the main bacterial nucleoid-associated protein HU. EMBO Rep..

[15] Hardy C.D., Cozzarelli N.R. (2005). A genetic selection for supercoiling mutants of Escherichia coli reveals proteins implicated in chromosome structure. Mol. Microbiol..

[16] Macvanin M., Adhya S. (2012). Architectural organisation in E. coli nucleoid. Biochim. Biophys. Acta.

[17] Ohniwa R.L., Muchaku H., Saito S., Wada C., Morikawa K. (2013). Atomic force microscopy analysis of the role of major DNA-binding proteins in organization of the nucleoid in Escherichia coli. PLoS One.

[18] Nielsen H.J., Ottesen J.R., Youngren B., Austin S.J., Hansen F.G. (2006). The Escherichia coli chromosome is organized with the left and right chromosome arms in separate cell halves. Mol. Microbiol..

[19] Wang X., Liu X., Possoz C., Sherratt D.J. (2006). The two Escherichia coli chromosome arms locate to separate cell halves. Genes Dev..

[20] Reyes-Lamothe R., Possoz C., Danilova O., Sherratt D.J. (2008). Independent positioning and action of Escherichia coli replisomes in live cells. Cell.

[21] Wiggins P.A., Cheveralls K.C., Martin J.S., Lintner R., Kondev J. (2010). Strong intranucleoid interactions organize the Escherichia coli chromosome into a nucleoid filament. Proc. Natl Acad. Sci. U.S.A..

[22] Vora T., Hottes A.K., Tavazoie S. (2009). Protein occupancy landscape of a bacterial genome. Mol. Cell.

[23] Montero Llopis P., Jackson A.F., Sliusarenko O., Surovtsev I., Heinritz J., Emonet T., Jacobs-Wagner C. (2010). Spatial organization of the flow of genetic information in bacteria. Nature.

[24] Kuhlman T.E., Cox E.C. (2012). Gene location and DNA density determine transcription factor distributions in Escherichia coli. Mol. Syst. Biol..

[25] Bryant J.A., Sellars L.E., Busby S.J., Lee D.J. (2015). Chromosome position effects on gene expression in Escherichia coli K-12. Nucleic Acids Res..

[26] Ball C.A., Osuna R., Ferguson K.C., Johnson R.C. (1992). Dramatic changes in Fis levels upon nutrient upshift in Escherichia coli. J. Bacteriol..

[27] Ninnemann O., Koch C., Kahmann R. (1992). The Ecoli fis promoter is subject to stringent control and autoregulation. EMBO J..

[28] Nilsson L., Verbeek H., Vijgenboom E., van Drunen C., Vanet A., Bosch L. (1992). FIS-dependent trans activation of stable RNA operons of Escherichia coli under various growth conditions. J. Bacteriol..

[29] Gonzalez-Gil G., Bringmann P., Kahmann R. (1996). FIS is a regulator of metabolism in Escherichia coli. Mol. Microbiol..

[30] Schneider R., Travers A., Kutateladze T., Muskhelishvili G. (1999). A DNA architectural protein couples cellular physiology and DNA topology in Escherichia coli. Mol. Microbiol..

[31] Blot N., Mavathur R., Geertz M., Travers A., Muskhelishvili G. (2006). Homeostatic regulation of supercoiling sensitivity coordinates transcription of the bacterial genome. EMBO Rep..

[32] Bradley M., Beach M.B., de Koning A.P.J., Pratt T.S., Osuna R. (2007). Effects of Fis on Escherichia coli gene expression during different growth stages. Microbiology.

[33] Schneider R., Travers A., Muskhelishvili G. (1997). FIS modulates growth-phase-dependent topological transitions of DNA in Escherichia coli. Mol. Microbiol..

[34] Keane O.M., Dorman C.J (2003). The gyr genes of Salmonella enterica serovar Typhimurium are repressed by the factor for inversion stimulation Fis. Mol. Genet. Genomics.

[35] Grainger D.C., Goldberg M.D., Lee D.J., Busby S.J. (2008). Selective repression by Fis and H-NS at the Escherichia coli dps promoter. Mol. Microbiol..

[36] Martinez A., Kolter R. (1997). Protection of DNA during oxidative stress by the nonspecific DNA-binding protein Dps. J. Bacteriol..

[37] Bochner B.R., Gadzinski P., Panomitros E. (2001). Phenotype microarrays for high-throughput phenotypic testing and assay of gene function. Genome Res..

[38] Hansen S., Lewis K., Vulić M. (2008). Role of global regulators and nucleotide metabolism in antibiotic tolerance in Escherichia coli. Antimicrob. Agents Chemother..

[39] Pratt T.S., Steiner T., Feldman L.S., Walker K.A., Osuna R. (1997). Deletion analysis of the fis promoter region in Escherichia coli, antagonistic effects of integration host factor and Fis. J. Bacteriol..

[40] Nasser W., Schneider R., Travers A., Mushkelishvili G. (2001). CRP modulates fis transcription by alternate formation of activating and repressing nucleoprotein complexes. J. Biol. Chem..

[41] Nasser W., Rochman M., Muskhelishvili G. (2002). Transcriptional regulation of the fis operon involves a module of multiple coupled promoters. EMBO J..

[42] Crozat E., Winkworth C., Gaffé J., Hallin P.F., Riley M.A., Lenski R.E., Schneider D. (2010). Parallel genetic and phenotypic evolution of DNA superhelicity in experimental populations of Escherichia coli. Mol. Biol. Evol..

[43] Nafissi M., Chau J., Xu J., Johnson R.C. (2012). Robust translation of the nucleoid protein Fis requires a remote upstream AU element and is enhanced by RNA secondary structure. J. Bacteriol..

[44] Gerganova V., Maurer S., Stoliar L., Japaridze A., Nasser W., Kutateladze T., Travers A., Muskhelishvili G. (2015). Upstream binding of idling RNA polymerase modulates transcription initiation from a nearby promoter. J. Biol. Chem..

[45] Schneider R., Travers A., Muskhelishvili G. (2000). The expression of the Escherichia coli fis gene is strongly dependent on the superhelical density of DNA. Mol. Microbiol..

[46] Jauregui R., Abreu-Goodger C., Moreno-Hagelsieb G., Collado-Vides J., Merino E. (2003). Conservation of DNA curvature signals in regulatory regions of prokaryotic genes. Nucleic Acids Res..

[47] Cameron A.D., Kröger C., Quinn H.J., Scally I.K., Daly A.J., Kary S.C., Dorman C.J. (2013). Transmission of an oxygen availability signal at the Salmonella enterica serovar Typhimurium fis promoter. PLoS One.

[48] Muskhelishvili G., Travers A., Dame R, Dorman C (2010). FIS and nucleoid dynamics upon exit from lag phase. Bacterial Chromatin.

[49] von Meyenburg K., Hansen F.G., Neidhardt FC (1987). Escherichia coli and Salmonella typhimurium. Cellular and Molecular Biology.

[50] Condon C., French S., Squires C., Squires C.L. (1993). Depletion of functional ribosomal RNA operons in Escherichia coli causes increased expression of the remaining intact copies. EMBO J..

[51] Dennis P.P., Ehrenberg M., Bremer H. (2004). Control of rRNA synthesis in Escherichia coli, a systems biology approach. Microbiol. Mol. Biol. Rev..

[52] Condon C., Philips J., Fu Z.Y., Squires C., Squires C.L. (1992). Comparison of the expression of the seven ribosomal RNA operons in Escherichia coli. EMBO J..

[53] Brambila E., Sclavi B. (2015). Gene regulation by H-NS as a function of growth conditions depends on chromosomal position in Escherichia coli. G3 (Bethesda).

[54] Ohniwa R., Morikava K., Kim J., Ohta T., Ishihama A., Wada C., Takeyasu K. (2006). Dynamic state of DNA topology is essential for genome condensation in bacteria. EMBO J..

[55] Nair S., Finkel S.E. (2004). Dps protects cells against multiple stresses during stationary phase. J. Bacteriol..

[56] Dersch P., Kneip S., Bremer E. (1994). The nucleoid-associated DNA-binding protein H-NS is required for the efficient adaptation of Escherichia coli K-12 to a cold environment. Mol. Gen. Genet..

[57] Nevo-Dinur K., Nussbaum-Shochat A., Ben-Yehuda S., Amster-Choder O (2011). Translation-independent localization of mRNA in E coli. Science.

[58] Muskhelishvili G., Travers A. (2013). Integration of syntactic and semantic properties of the DNA code reveals chromosomes as thermodynamic machines converting energy into information. Cell. Mol. Life Sci..

