# Supplementary material for: Chromosomal position shift of a regulatory gene alters the bacterial phenotype
Source: Nucleic Acids Res. 2015 Jul 13;43(17):8215–26. doi: 10.1093/nar/gkv709 (PMC4751926; doi:10.1093/nar/gkv709)
Supplement: SUPPLEMENTARY DATA [file supp_43_17_8215__index.html]

Chromosomal position shift of a regulatory gene alters the bacterial phenotype — Chromosomal position shift of a regulatory gene alters the bacterial phenotype — SUPPLEMENTARY DATA 

# Chromosomal position shift of a regulatory gene alters the bacterial phenotype

## SUPPLEMENTARY DATA

- SUPPLEMENTARY DATA
