# Supplementary material for: Chromosomal position shift of a regulatory gene alters the bacterial phenotype
Source: Nucleic Acids Res. 2015 Jul 13;43(17):8215–26. doi: 10.1093/nar/gkv709 (PMC4751926; doi:10.1093/nar/gkv709)
Supplement: SUPPLEMENTARY DATA [file supp_gkv709_nar-01616-x-2015-File008.pdf]

SUPPLEMENTARY FIGURES AND TABLES

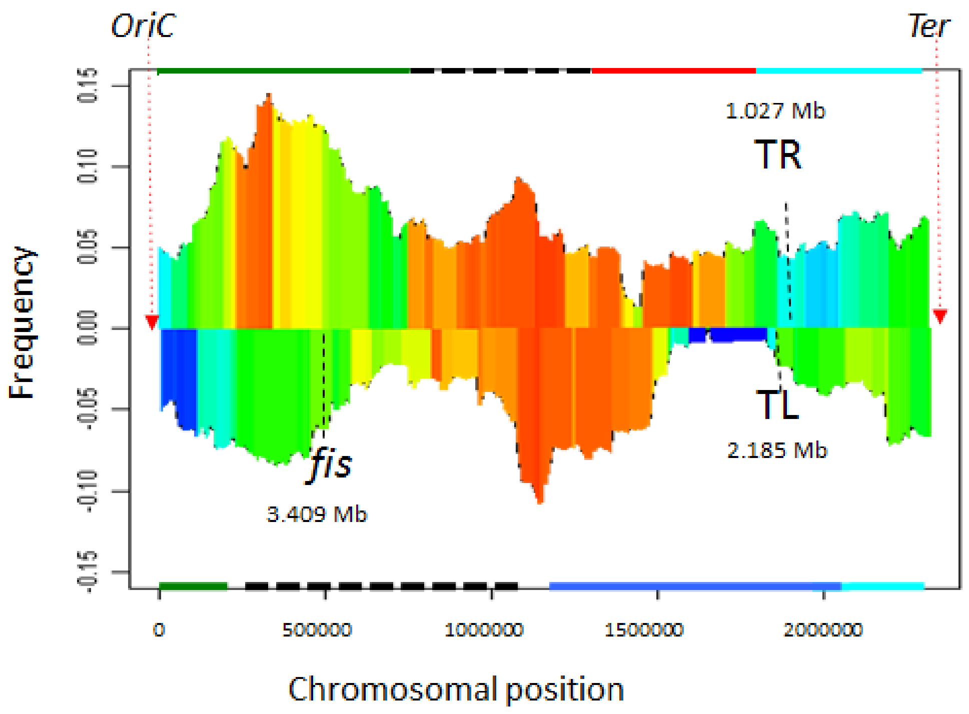

Figure S1. Distribution of the FIS binding sites in the genome (RegulonDB) calculated by using a sliding window of 400 kb and normalising over the total gene number for each window (modified from Sobetzko et al, 2012). The replichores are organised from *OriC* to *Ter* (left to right). Above and below the distributions the Ori, Ter, left and right macrodomains (green, cyan, blue and red lines respectively) are indicated on the chromosomal replichores. The frequency distributions (ordinate) for the right and the left replichore are respectively plotted above and below the zero on the ordinate. The original chromosomal position of *fis* and the insertions in TR and TL are indicated. The direction of the FIS effect is rainbow color-coded with blue for repression and red for activation.

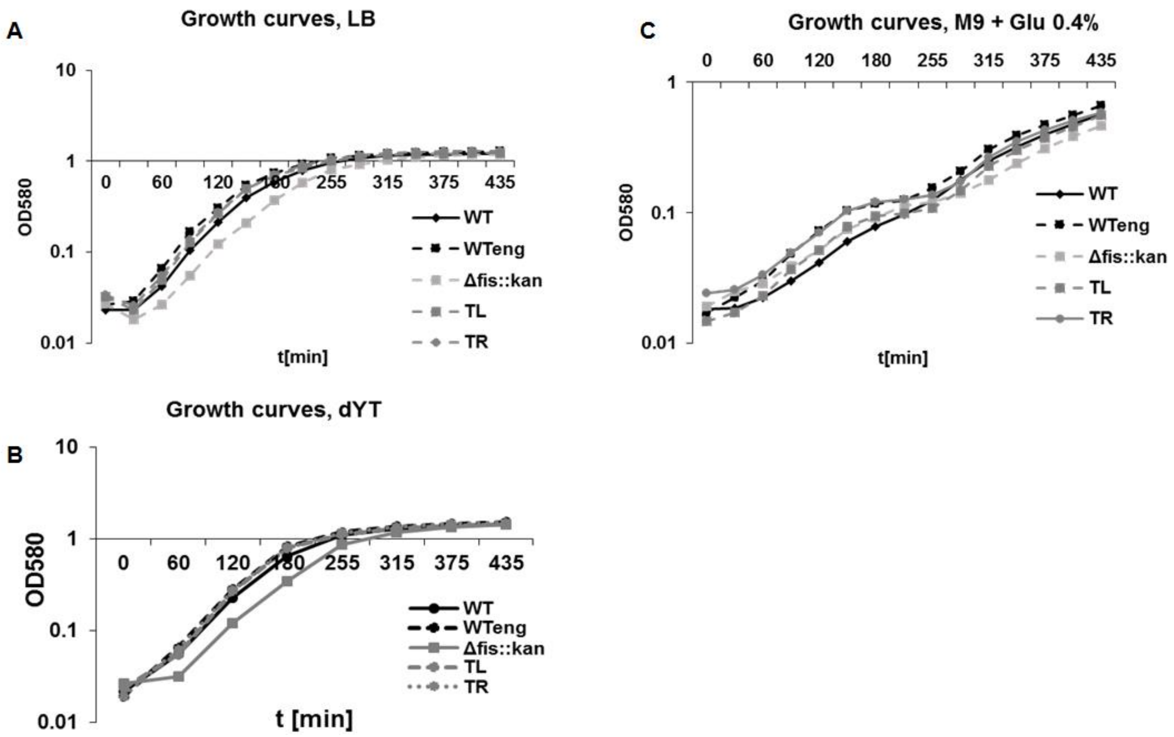

Figure S2. Growth curves of the movants and control strains in LB (A), dYT (B), and M9 supplemented with glucose (C). Abscissa – time (min) after inoculation. Ordinate – OD_{580}.

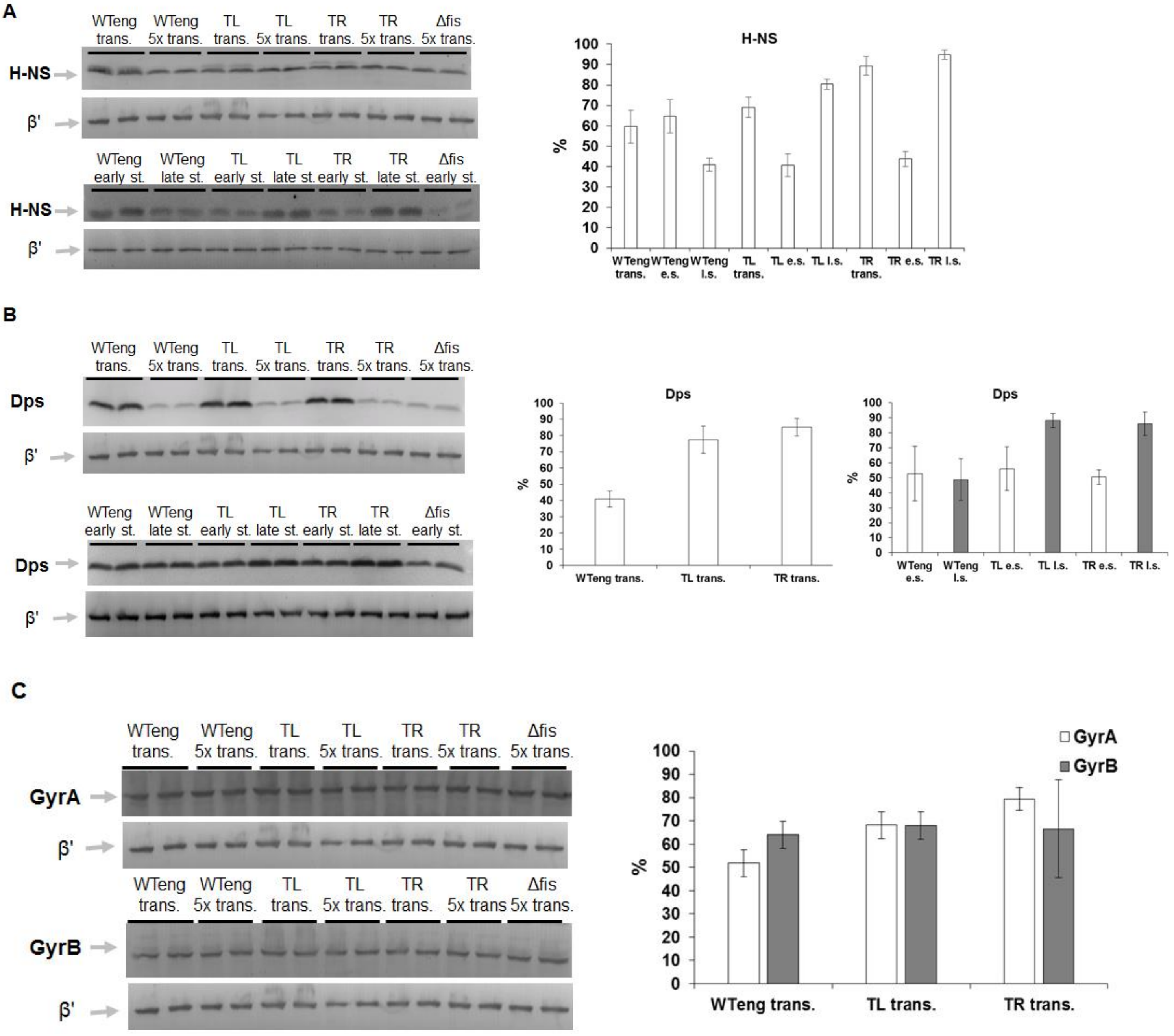

A
WTeng trans.
WTeng 5x trans.
TL trans.
TL 5x trans.
TR trans.
TR 5x trans.
Δfis 5x trans.
H-NS
β'
WTeng early st.
WTeng late st.
TL early st.
TL late st.
TR early st.
TR late st.
Δfis early st.
H-NS
β'
H-NS
%
100
90
80
70
60
50
40
30
20
10
0
WTeng trans.
WTeng e.s.
WTeng l.s.
TL trans.
TL e.s.
TL l.s.
TR trans.
TR e.s.
TR l.s.
B
WTeng trans.
WTeng 5x trans.
TL trans.
TL 5x trans.
TR trans.
TR 5x trans.
Δfis 5x trans.
Dps
β'
WTeng early st.
WTeng late st.
TL early st.
TL late st.
TR early st.
TR late st.
Δfis early st.
Dps
β'
Dps
%
WTeng trans.
TL trans.
TR trans.
Dps
%
WTeng e.s.
WTeng l.s.
TL e.s.
TL l.s.
TR e.s.
TR l.s.
C
WTeng trans.
WTeng 5x trans.
TL trans.
TL 5x trans.
TR trans.
TR 5x trans.
Δfis 5x trans.
GyrA
β'
WTeng trans.
WTeng 5x trans.
TL trans.
TL 5x trans.
TR trans.
TR 5x trans
Δfis 5x trans.
GyrB
β'
GyrA
GyrB
%
WTeng trans.
TL trans.
TR trans.

D

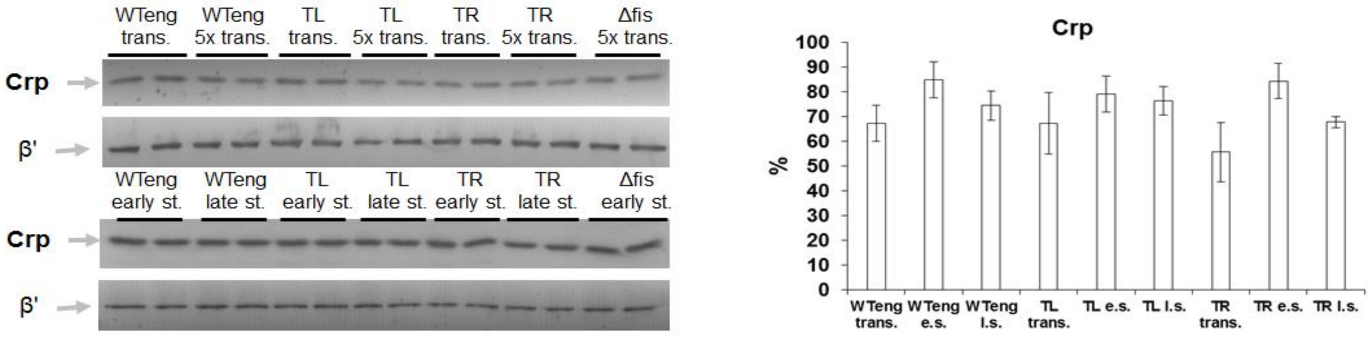

WTeng trans.
WTeng 5x trans.
TL trans.
TL 5x trans.
TR trans.
TR 5x trans.
Δfis 5x trans.
Crp
β'
WTeng early st.
WTeng late st.
TL early st.
TL late st.
TR early st.
TR late st.
Δfis early st.
Crp
β'
Crp
%
WTeng trans.
WTeng e.s.
WTeng l.s.
TL trans.
TL e.s.
TL l.s.
TR trans.
TR e.s.
TR l.s.

E

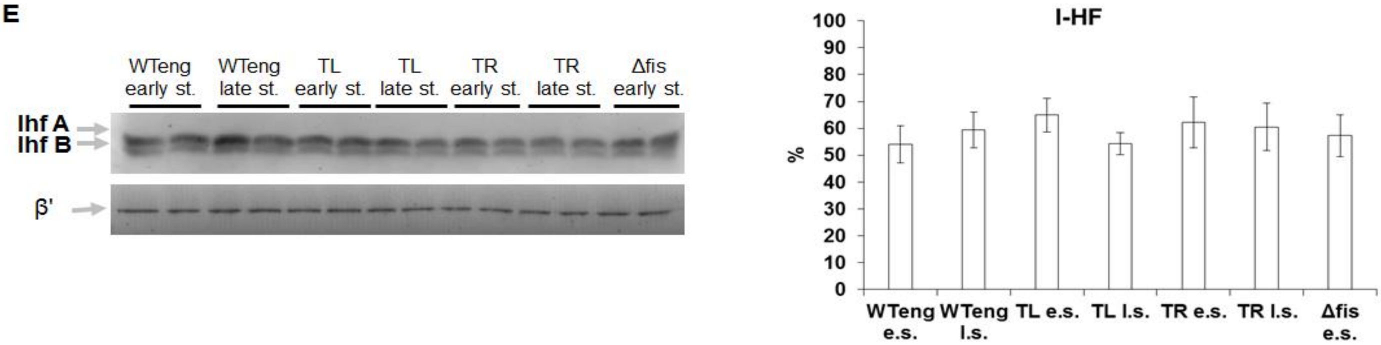

WTeng early st.
WTeng late st.
TL early st.
TL late st.
TR early st.
TR late st.
Δfis early st.
Ihf A
Ihf B
β'
I-HF
%
WTeng e.s.
WTeng l.s.
TL e.s.
TL l.s.
TR e.s.
TR l.s.
Δfis e.s.

F

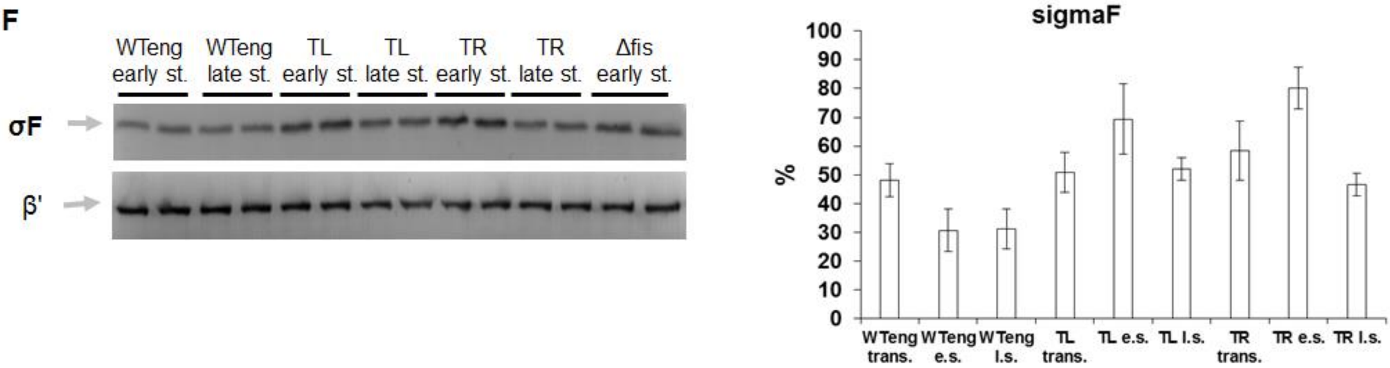

WTeng early st.
WTeng late st.
TL early st.
TL late st.
TR early st.
TR late st.
Δfis early st.
σF
β'
sigmaF
%
WTeng trans.
WTeng e.s.
WTeng l.s.
TL trans.
TL e.s.
TL l.s.
TR trans.
TR e.s.
TR l.s.

G

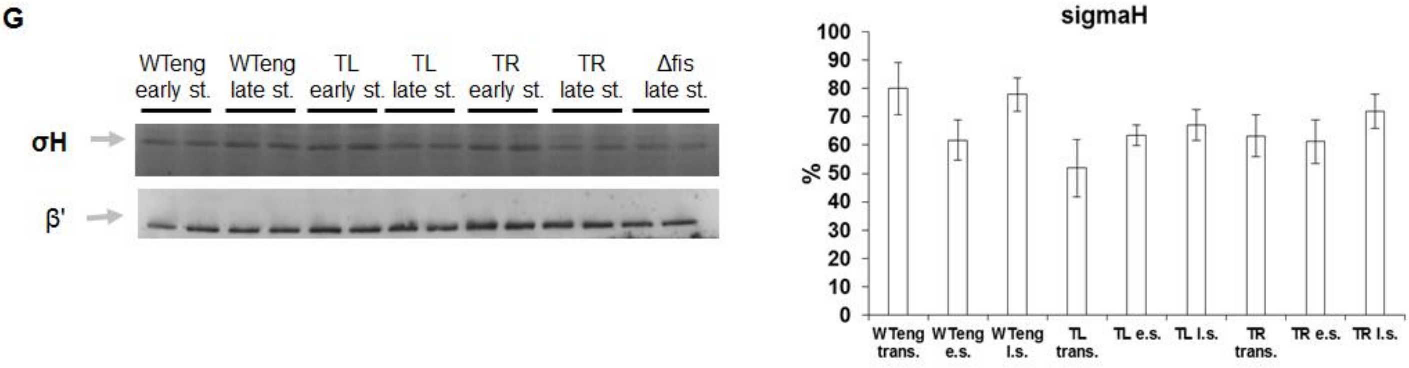

WTeng early st.
WTeng late st.
TL early st.
TL late st.
TR early st.
TR late st.
Δfis late st.
σH
β'
sigmaH
%
WTeng trans.
WTeng e.s.
WTeng l.s.
TL trans.
TL e.s.
TL l.s.
TR trans.
TR e.s.
TR l.s.

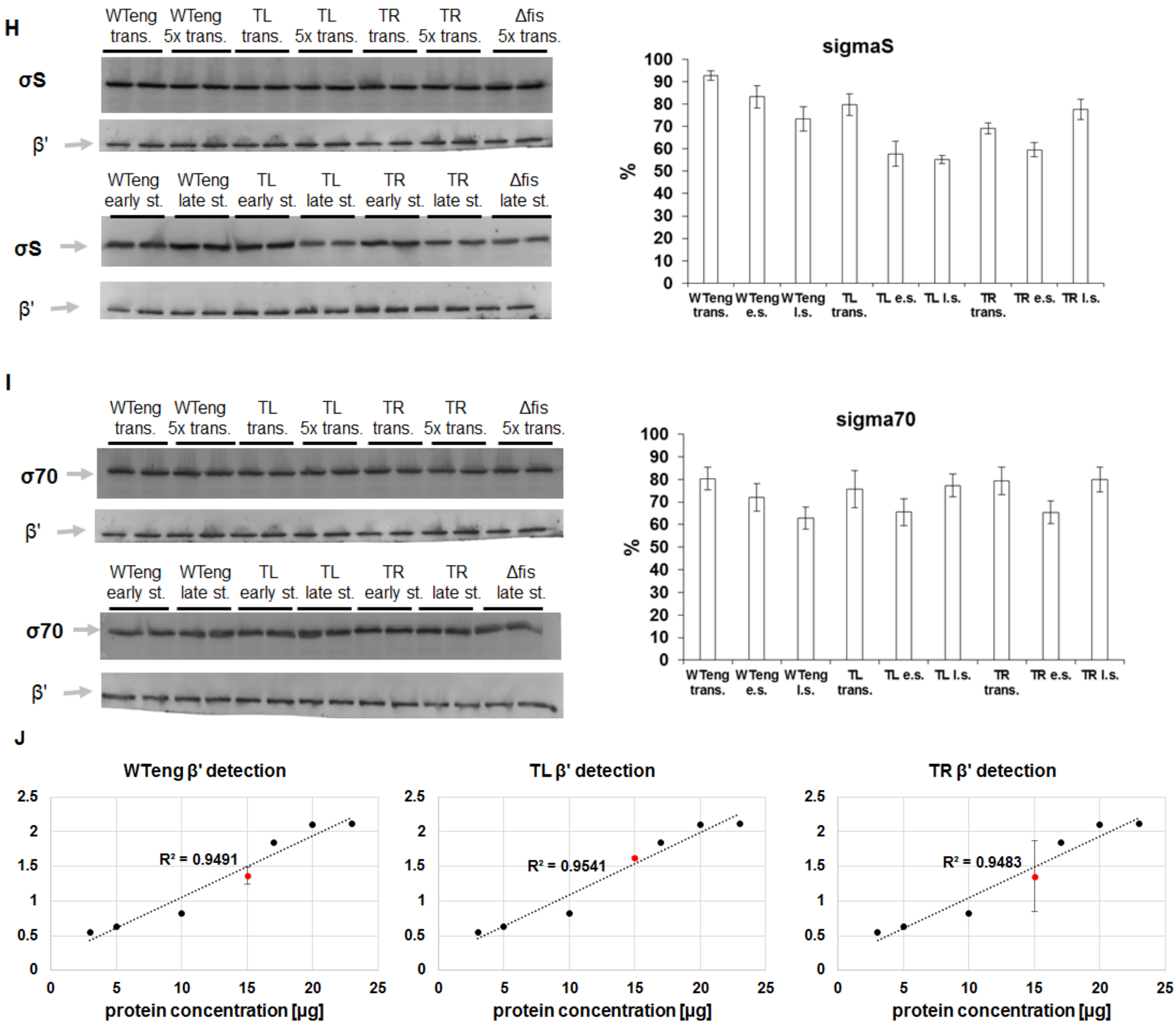

Figure S3. Western Blot gallery. (a-j) Representative blots are shown together with a respective RNA polymerase ß' subunit control used for normalisation. Quantification plots are presented alongside the blots for each detected protein. In order to allow for a quantitative comparison across the separate experiments, the normalized ratios were converted into percentages. AIDA software was used for quantification and Microsoft Excel was used for the further calculations. All error bars are standard errors. (a) H-NS; (b) Dps; (c) GyrA and GyrB; (d) CRP; (e) IHF; (f) sigmaF; (g) sigmaH; (h) sigmaS; (i) sigma70. The lanes labelled ''5x trans.'' are obtained from "multiple passage" experiment in which strains were grown to transition phase and re-diluted in fresh medium to test whether this procedure can affect the result. This cycle was repeated up to 5 times and named 5x trans. However only the data from single passage experiments was considered in quantifications. (j) Representative standard curve of samples with varying protein concentrations detected with ß' antibody shows that the selected loading concentration of 15 µg lies in a linear segment of the trendline. Similar curves have been determined for each of the used antibodies. Error bars are standard errors (some standard errors are too small to be visible).

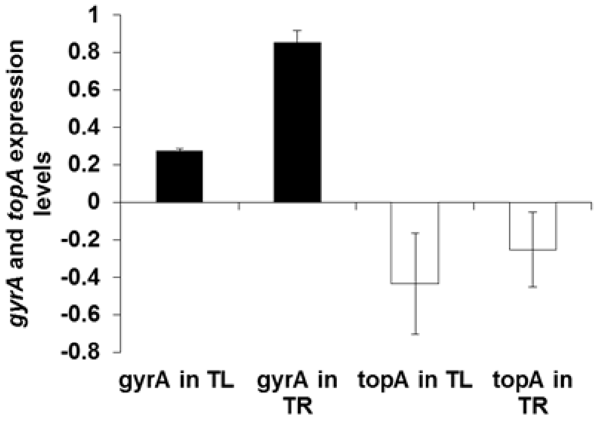

Figure S4. Quantitative analyses (qRT-PCR) of mRNA levels of *gyrA* and *topA* in the movant strains with WTeng, serving as baseline. The Y-axis is on a logarithmic scale (ln), such that a value of +1.0 and -1.0 respectively indicate a two-fold increase and decrease compared to the baseline.

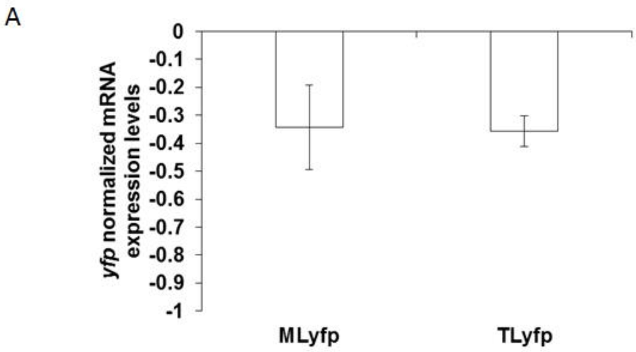

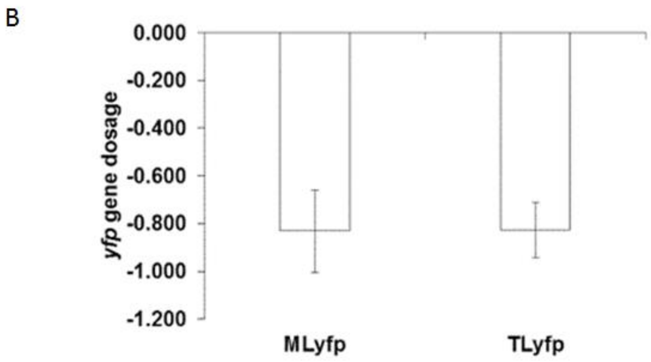

Figure S5. Expression levels of *yfp* mRNA (upper panel) and determination of *yfp* gene dosage (lower lanel) in middle left (MLyfp) and terminus left (TLyfp) *dusB-yfp-cat* insertion strains during early exponential growth phase, OD 0.3. The values determined for the *dusB-yfp-cat* construct inserted in the native *fis* locus serve as baseline. The Y-axis represents the *yfp* mRNA levels (upper panel) and gene dosage (lower panel) in logarithmic (ln) scale, such that a value of -1.0 entails a two-fold decrease compared to the baseline. The MLyfp and TLyfp are Δ*fis* strains to allow a valid comparison to the baseline strain, which carries the *yfp* reporter gene in the native locus, instead of *fis.* All error bars are standard errors.

Table S1. List of antibodies used in the study.

Primary Antibody	Working dilution	Manufacturer
Mouse anti β'	1:1000	NeoClone®
Rabbit anti GyrA	1:1000	Inspiralis Ltd
Mouse anti σ^F	1:1000	NeoClone®
Mouse anti σ^S	1:1000	NeoClone®
Mouse anti σ^H	1:1000	NeoClone®
Rabbit anti FIS	1:200	gift Christian Koch
Rabbit anti Dps	1:1000	gift Dominique Schneider
Rabbit anti H-NS	1:1000	gift Rolf Wagner
**Secondary Antibody**	**Working dilution**	**Manufacturer**
AP-Goat anti rabbit	1:30000	ZYMED® (Invitrogen™)
AP-Goat anti mouse	1:1000	Cell Signaling Technology, INC

Table S2. List of primers used in the study.

	Primer	Sequence 5' -> 3'
1	WTeng	5´- ttcaggttagctaaatgcttgattaaaaaggcgctactcggcatggggatggagaaaaaaatcactgg-3´
2	WTeng	5'-catcctgttctcatggtcactccctttgtgacacctataaaaaaggcgcttacgccccgccctgccactc-3'
3	TL for	5'- caacgtgaaatacgactaataacaagcaagacgagcaagtggctaataatccggtcgtaagaattaacct-3'
4	TL rev	5'-atcatcgctaaaaaaagccccctcatcatgaggggaaatgcagacacctttacgccccgccctgccactc-3'
5	TR for	5'-attgagatccctcgcctgggcctggccaaataaaaaatccccggaaggcaccggtcgtaagaattaacct-3'
6	TR rev	5'-aaacaactccaggccccgcgtctgcgtaactaatccctgaacaaatccccttacgccccgccctgccactc-3'
7	ML for	5'-gttttgcttggtgaatggtggcgtcagaataaagcctgataaatcagccgccggtcgtaagaattaacct-3'
8	ML rev	5'-agtattaccggcagagagtgagtaaatttgcggggaaatgccggatggcattacgccccgccctgccactc-3'
9	yohN (rcnB) for	cgggttcgaccaatacctcg
10	yohN (rcnB) rev	cacccatataggtccagtgcg
11	yehA for	gtgcttcatcatcgacacgc
12	yehA rev	cctgcattttgtgccaatgc
13	yccU for	cagaaggggtatggcacgct
14	yccU rev	catcccgtgccagtactgcc
15	yccV (hspQ) for	caggtccgccattccctgtt
16	yccV (hspQ) rev	cggtaggccgttatcgtcct
17	fis for	tgctcaactgaatggtcagg
18	fis rev	ggttaccacgggtgtattgc
19	topA for	aggcgcactatgaagtgttg
20	topA rev	tcggttgcgagatagatgtg
21	gyrA for	aggtatggcaaccaacatcc
22	gyrA rev	atgtgttccatcagcccttc
23	yfp for	cgtgaccaccttcggctac
24	yfp rev (mRNA)	gaagatggtgcgctcctg
25	yfp rev (gene dosage)	ttacttgtacagctcgtcca

Table S3. *E.coli* strains used in the study.

Strain	Description	Origin
**CSH50**	*E.coli* K12 strain CSH50 (*ara D(lac pro) thi rpsL)*	Miller (1972)*
**WTeng**	A derivative of CSH50 with a *cat* cassette introduced after the *fis* gene.	This study.
**CSH50 Δ*fis::kan***	A derivative of CSH50 with *kan* cassette disrupting the *fis* ORF.	This study.
**TL**	A derivative of CSH50 Δ*fis::kan* with *fisP-dusB-fis-cat* introduced between pos. 2,185,320 and 2,185,402.	This study.
**TR**	A derivative of CSH50 Δ*fis::kan* with *fisP-dusB-fis-cat* introduced between pos. 1,027,582 and 1,027,627.	This study.
**WTeng-yfp**	A derivative of CSH50 with *yfp* reporter gene replacing *fis* in its native locus, comprising a *fis*⁻ background.	Alissa Respet.
**TLyfp**	A derivative of CSH50 WT with *fisP-dusB-yfp-cat* introduced between pos. 2,185,320 and 2,185,402.	This study.
**TLyfp Δ*fis::kan***	A derivative of TLyfp with *fis* gene deleted from its native locus. Generated via classical P1 transduction.	This study.
**TRyfp**	A derivative of CSH50 WT with *fisP-dusB-yfp-cat* introduced between pos. 1,027,582 and 1,027,627.	This study.
**TRyfp Δ*fis::kan***	A derivative of TRyfp with *fis* gene deleted from its native locus. Generated via classical P1 transduction.	This study.
**MLyfp**	A derivative of CSH50 WT with *fisP-dusB-yfp-cat* introduced between pos. 2,715,465 and 2,1715,513.	This study.
**MLyfp Δ*fis::kan***	A derivative of MLyfp with *fis* gene deleted from its native locus. Generated via classical P1 transduction.	This study.

*Miller, J. H. (1972). Experiments in Molecular Genetics, Cold Spring Harbor Laboratory Press, New York.
